# Supplementary material for: Single-virus genomics reveals hidden cosmopolitan and abundant viruses
Source: Nat Commun. 2017 Jun 23;8:15892. doi: 10.1038/ncomms15892 (PMC5490008; doi:10.1038/ncomms15892)
Supplement: Supplementary Information [file ncomms15892-s1.pdf]

Title of file for HTML: Supplementary Information
Description: Supplementary Figures, Supplementary Tables, Supplementary Notes, Supplementary Methods and Supplementary References

Title of file for HTML: Peer Review File
Description:

Supplementary Information

Supplementary Figures

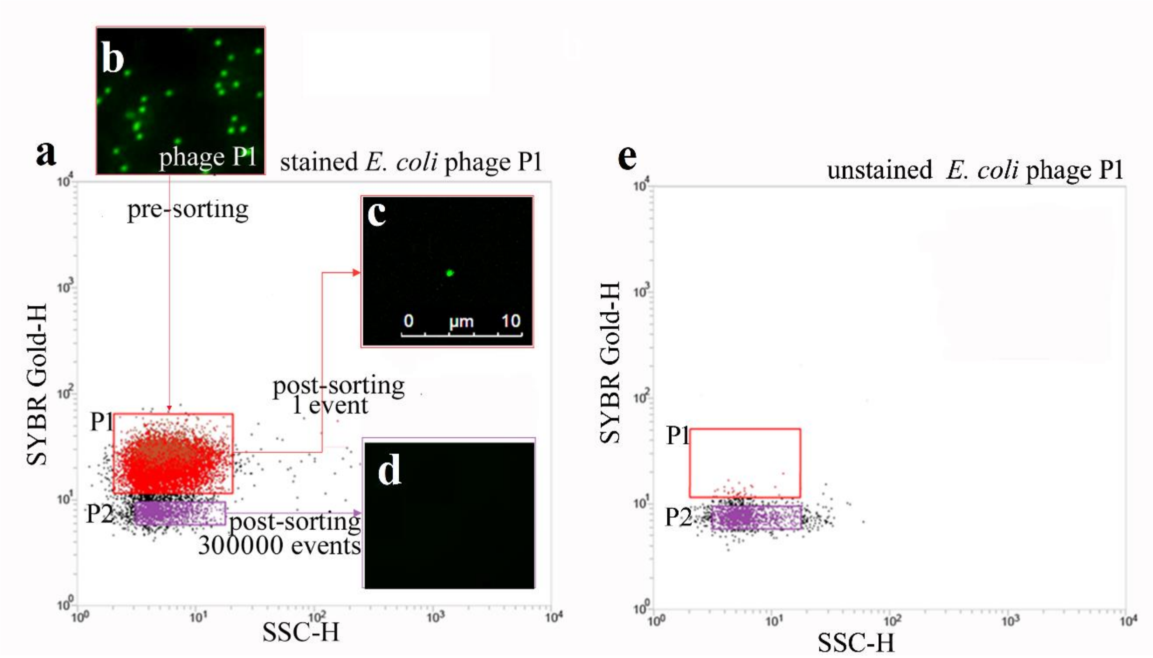

Supplementary Fig. 1. Fluorescence-activated virus sorting (FAVS) of bacteriophage P1 of *Escherichia coli*. (**a**) Flow cytometric plot of 90° light (side) scatter (SSC-H; height value) vs. green fluorescence after staining with SYBR Gold, (SYBR Gold-H; height value, relative units) of *E. coli* phage P1. Selected sorting gate of individual viral particles is indicated in red (gate P1). Background noise, gate P2. **(b)** Epifluorescence microscopy image of phage P1 culture used for sorting (pre-FAVS). **(c)** Confocal laser scanning microscopy of 1 sorted individual virus (post-sorting). A thorough scan was performed to rule out the presence of doublets or more coincident events. The experiment was repeated five times with identical results. **(d)** Epifluorescence image of 300,000 sorted events from background noise (gate P2). No stained viruses were detected in this area. **(e)** Flow cytometric plot of the unstained phage P1 (blank control).

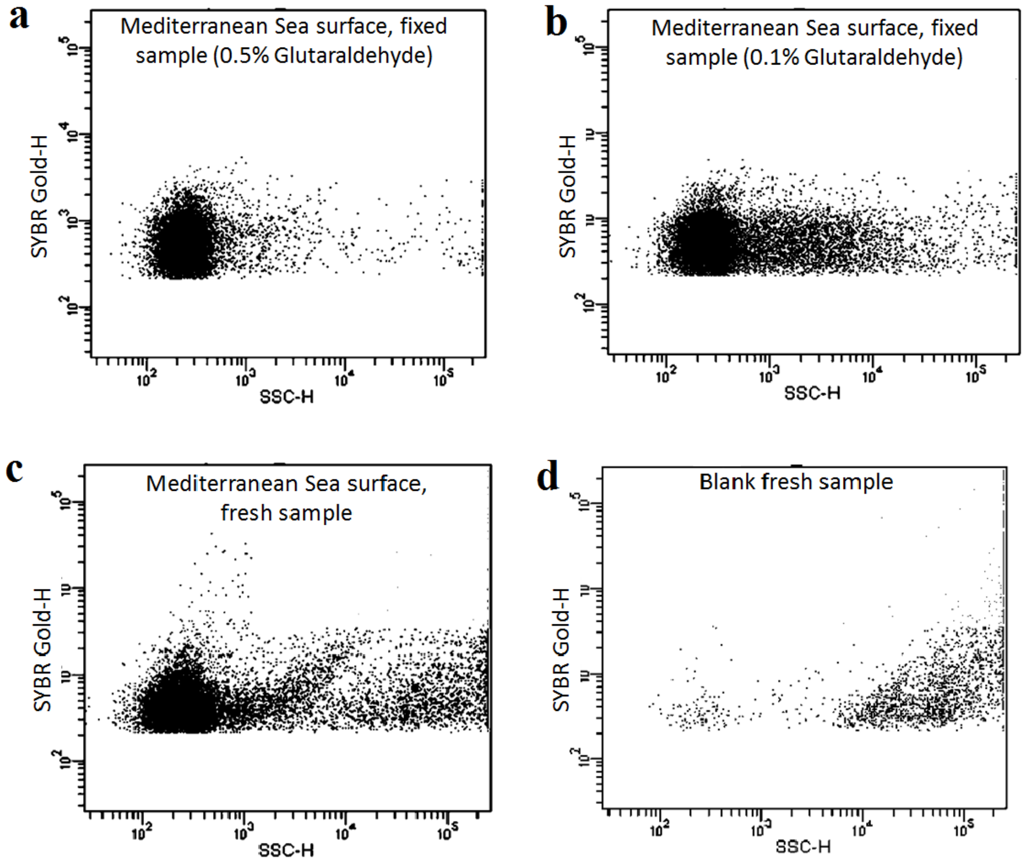

Supplementary Fig. 2. Virus staining optimization for fluorescence-activated virus sorting (FAVS). The standard and reference protocol used for staining and detection of viruses by flow cytometry for aquatic samples was that previously published by Corina Brussard[1]. However, the amount of fixative (0.5% glutaraldehyde) used in that protocol prevent the amplification of genetic material by multiple displacement amplification (MDA) and consequently subtle variations on that protocol were performed (see methods). Comparison of the staining of same marine viral samples with different fixation treatments (see Methods for details): fixed with 0.5% (panel **a;** reference protocol by Corina Brussard[1]), with 0.1% glutaraldehyde **(b)**, and fresh (unfixed) sample (**c**). Samples were stained with SYBR Gold 0.5X final concentration (see Methods for details). Flow cytometry was performed using FACS Canto II (see Methods). Our results indicated that the staining procedures used in this study showed similar results than the reference protocol traditionally used in viral ecology to count and detect viruses from natural marine samples.

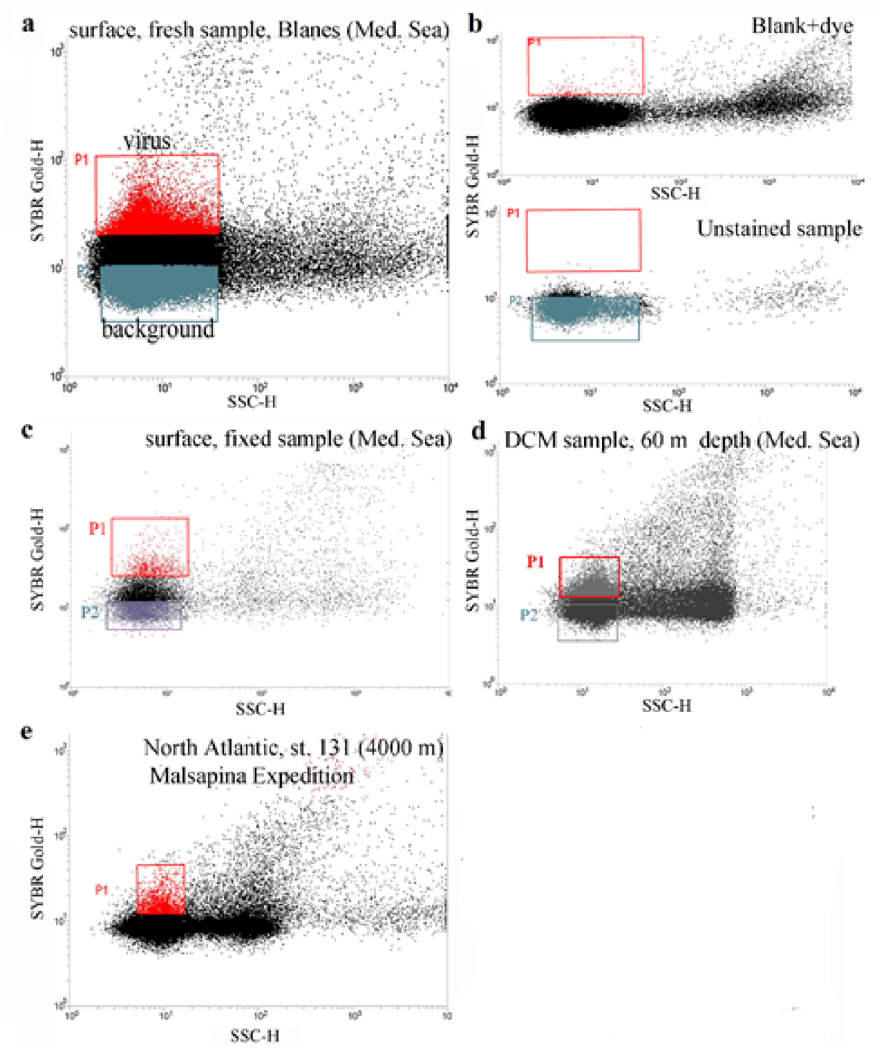

Supplementary Fig. 3. Fluorescence-activated virus sorting (FAVS) of marine and human salivary samples. For each sample, flow cytometric plot of 90° light scatter (SSC-H; height value) and green fluorescence, (SYBR Gold-H; height value, relative units) is shown. Gate P1 was used for sorting of single-viruses. **(a)** Surface seawater sample from the Blanes Bay Microbial Observatory (BBMO, Spain) in the Mediterranean Sea. **(b)** Blank and unstained viral fraction for the BBMO sample. No fluorescence signal was observed in gate P1. For all marine samples data from negatives were very similar. For convenience only negative and blank data are shown for BBMO. **(c)** Surface seawater sample from the Barcelona Beach (Barcelona, Spain) from the Mediterranean Sea. **(d)** Seawater sample from the deep chlorophyll maximum zone in the Mediterranean Sea (depth 60 m). **(e)** Deep seawater samples from the North Atlantic (4,000 m depth). Station 131 from the Malaspina Expedition. The deep seawater sample from station 134 showed a similar flow cytometric pattern.

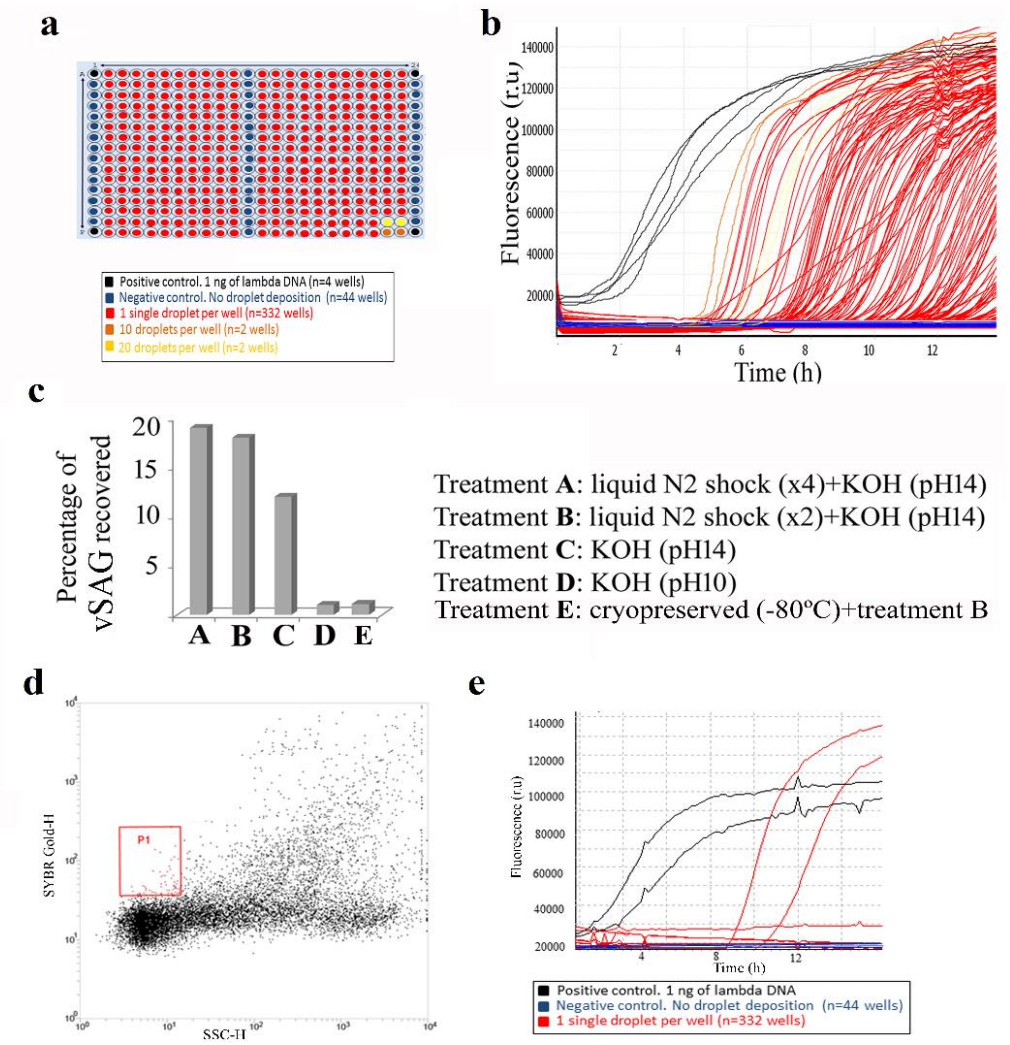

Supplementary Fig. 4. **Whole genome amplification (WGA) of marine single-viruses and assessment of effect of free DNA in seawater on WGA**. **(a)** Layout of a 384-well plate indicating the wells distribution. **(b)** Real-time multiple displacement amplification (MDA) of the genome of sorted single-viruses from the Blanes Bay Microbial Observatory (BBMO). **(c)** Efficiency of vSAGs recovery according to the various methods employed to break the capsid, with different cycles of freezing in liquid nitrogen followed by a shock in buffer KOH (pH 10 or 14). **(d** and **e)** Assessment of contribution of free DNA in to whole genome amplification of sorted single-viruses. **(d)** Flow cytometric plot of 90° light scatter (SSC-H; height value) and green SYBR Gold-H fluorescence (relative units, height values) of a stained seawater sample (Barcelona Beach in the Mediterranean Sea) previously filtered through 0.02 µm pore size to remove viruses. Putative free DNA was stained with SYBR Gold and processed as a fresh sample (see methods for details). Note that, as expected, stained putative free DNA were not detected in gate P1 used previously for virus sorting. Gate P1 was restricted for those events with higher fluorescence signals, which in theory would represent large stained free DNA fragments. **(e)** Real-time MDA results of sorted events with putative free DNA molecules deposited in a 384-well plate.

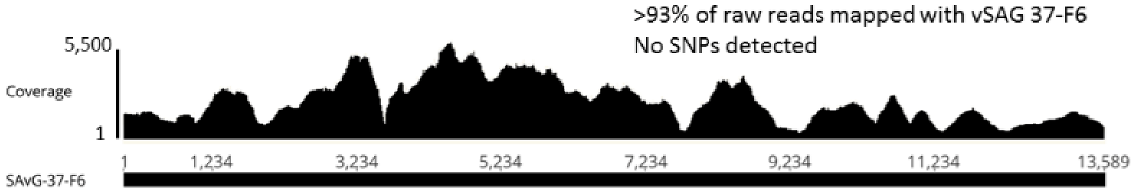

Supplementary Figure 5: **Raw Illumina reads mapping against the assembled genome of vSAG 37-F6**. Nearly all obtained reads for vSAG 37-F6 mapped perfectly without SNPs with the reconstructed genome indicating that the MDA did not generated chimeric artifacts. Only in two vSAGs, the 17-D19 and 41-A4, we observed that for each one, two assembled genome fragments with similar size were obtained with a similarity between 85 and 71%, respectively. We speculate that in this case, two viral particles from same population could be co-sorted. In the case of vSAG 17-D19, both genome fragments belonged to same viral cluster (see Supplementary Table 3).

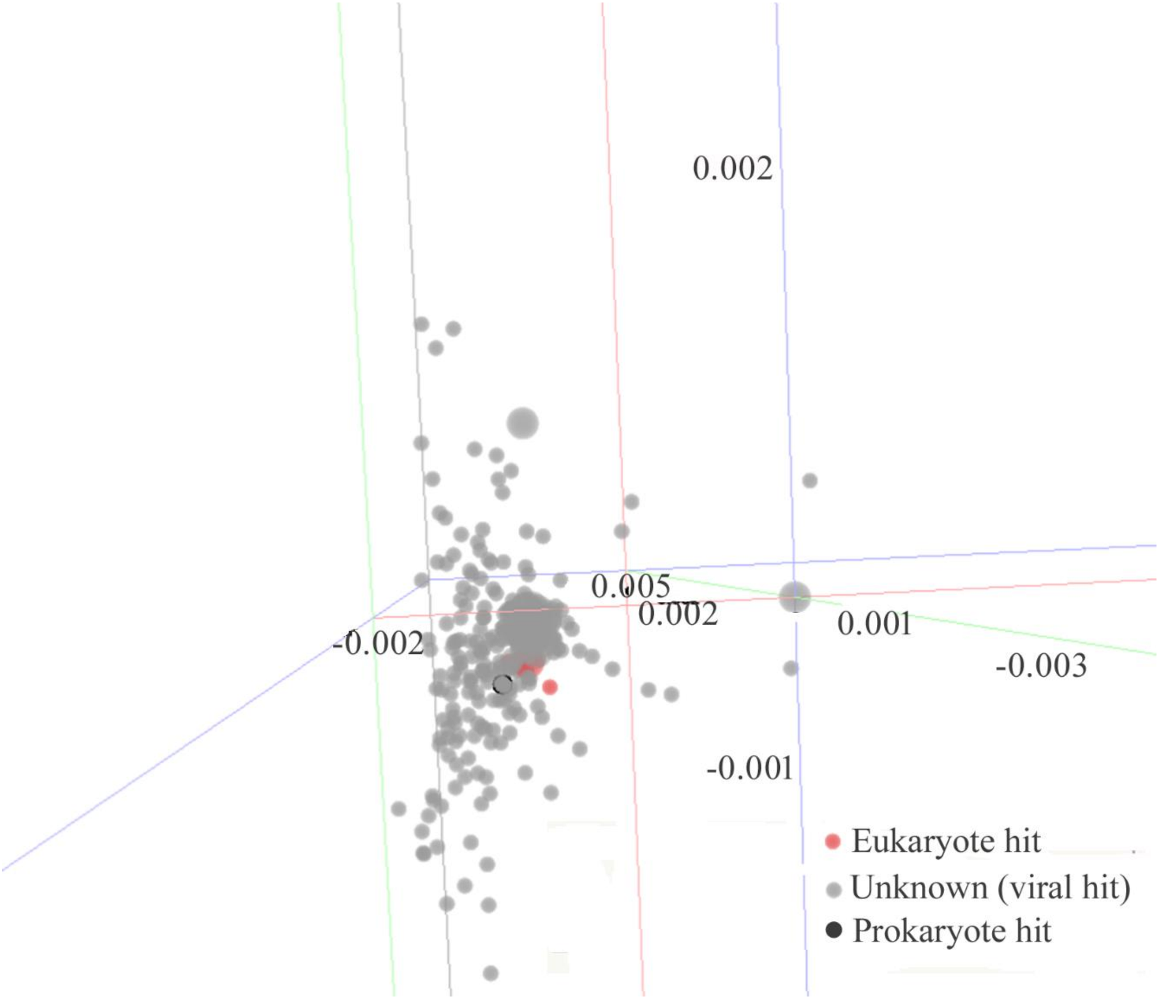

Supplementary Figure 6: Decontamination of genomic data of single amplified viral genomes. Decontamination was done by a semi-automatic approach by combining the use of the ProDeGe pipeline[2] and a thorough manual decontamination by BLASTx and BLASTn against the nr database. Detected contaminant contigs (typically <1kb length) were removed and the remained putative viral genome fragments were screened with ProDeGe pipeline and the results of the principal component analyses is shown. ProDeGe bins kmers (5-mers and 9-mers) generated from cleaned vSAGs and compare them by BLAST against nr Genbank database. Nearly all cleaned putative viral genome fragments were of unknown origin, taxonomically not related to prokaryotes. Each dot is a putative viral genome fragment. Color of dot indicates the putative taxonomic affiliation of the best hit kmers generated from vSAGS with the nr Genbank database.

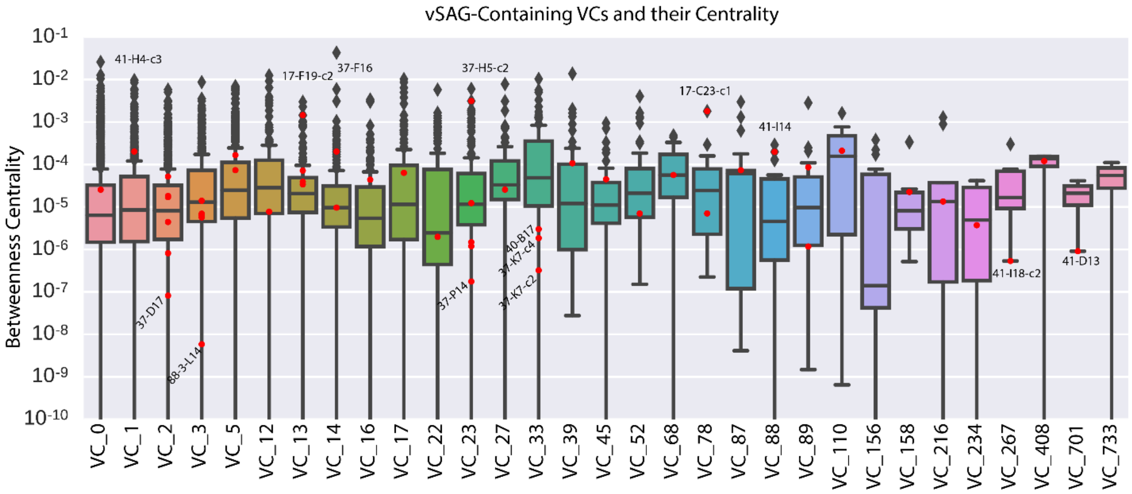

Supplementary Fig. 7. Relatedness of vSAGs with the Global Oceanic Virome clusters described in this study (Supplementary Table 3). Figure illustrates the centrality and frequency of connections between vSAGs and viral clusters (VCs, X-axis). Low betweenness values (Y-axis) correspond to fewer/weaker connections with VCs, with higher values being more-connected sequences. Each box represents 95% confidence intervals, with average score centrality within VCs denoted by a line in the box. vSAGs outliers (in red) below average score centrality could represent new genera. Although application of viral taxonomy criteria to define viral species and genera remains complicated to uncultured viruses, in this study we have used the following criteria based on a previous study by Roix and colleagues[4]. New genera are defined when the vSAGs presented weaker connections with closest viral relatives within the global marine viral network, as previously described[4]. New viral species are defined when ≤95% of nucleotide identity was obtained with the closest viral relative.

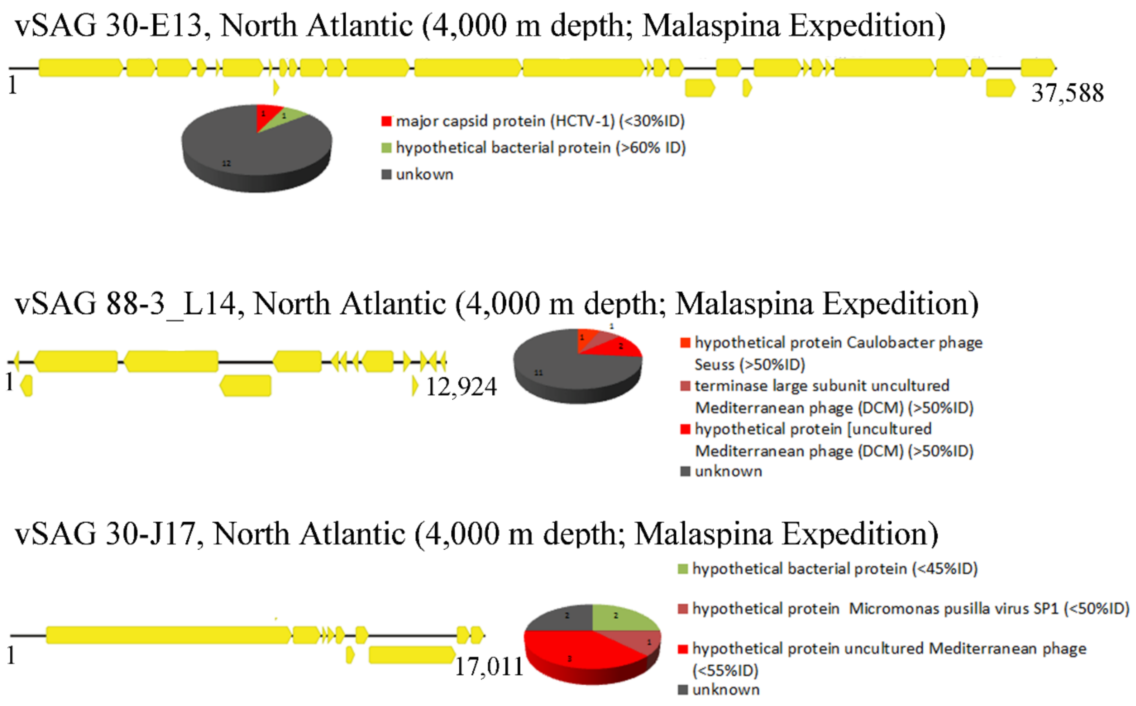

Supplementary Figure 8: Single amplified viral genomes obtained from the deep ocean. Genome annotation of three vSAGs from the North Atlantic. Prediction of open reading frames (ORFs) were done with Genmark with heuristic model optimized for viruses[3,4]. Comparison with BLASTp of predicted ORFs was carried out with non-redundant Genbank and viral fosmids from mesopelagic and bathypelagic samples of the Mediterranean Sea[5]. Conserved domains of predicted proteins were searched[6].

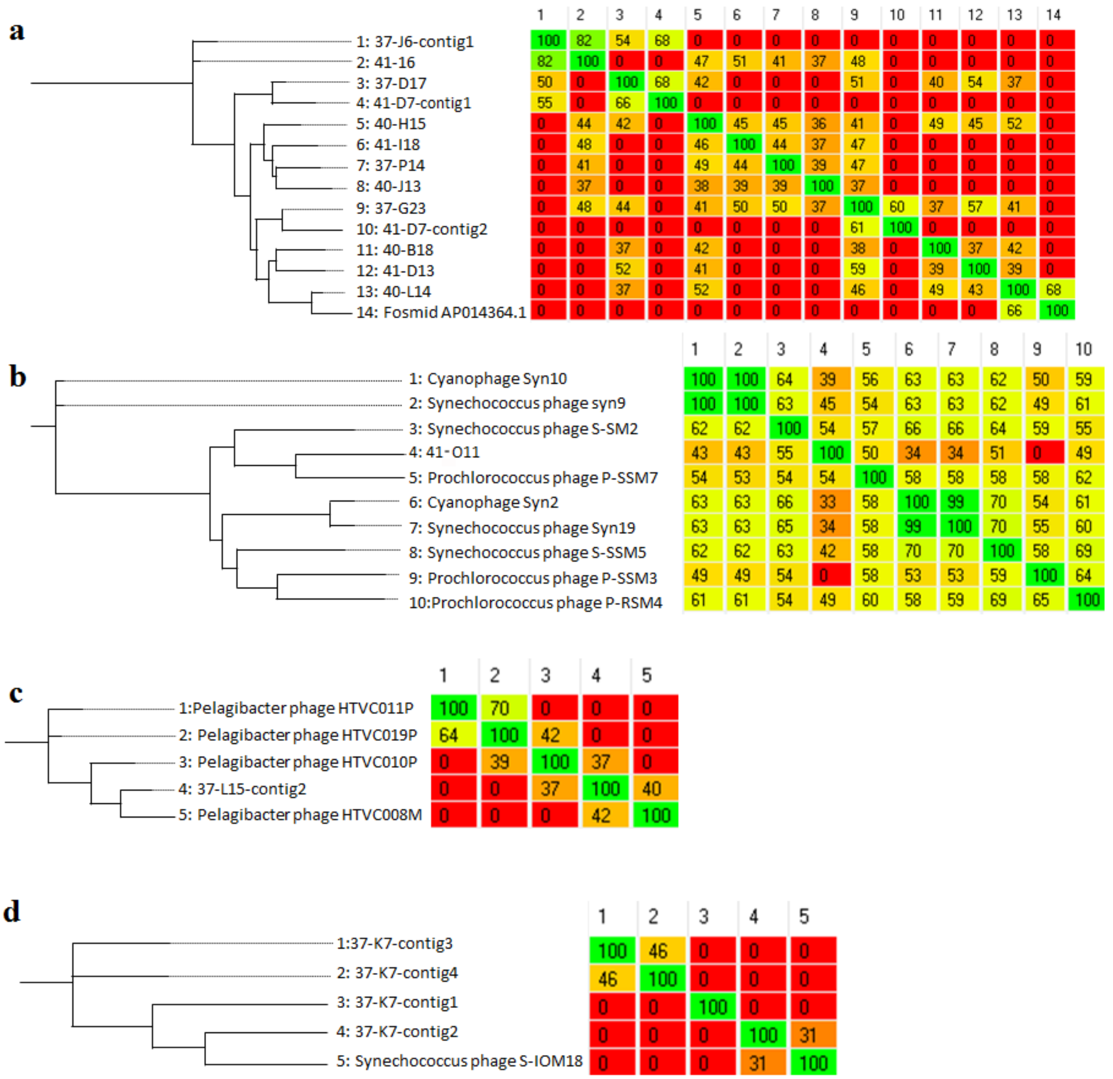

Supplementary Figure 9: Comparative genome analyses based on average nucleotide identity (ANI). **(a-d)** Different heat maps calculated using Gegenees 2.2.1 software showing the genetic relatedness (ANI values) within the obtained vSAGs **(a)**, and with other marine viral groups **(b-d)**. The trees were constructed with SplitsTree using the neighbor joining method.

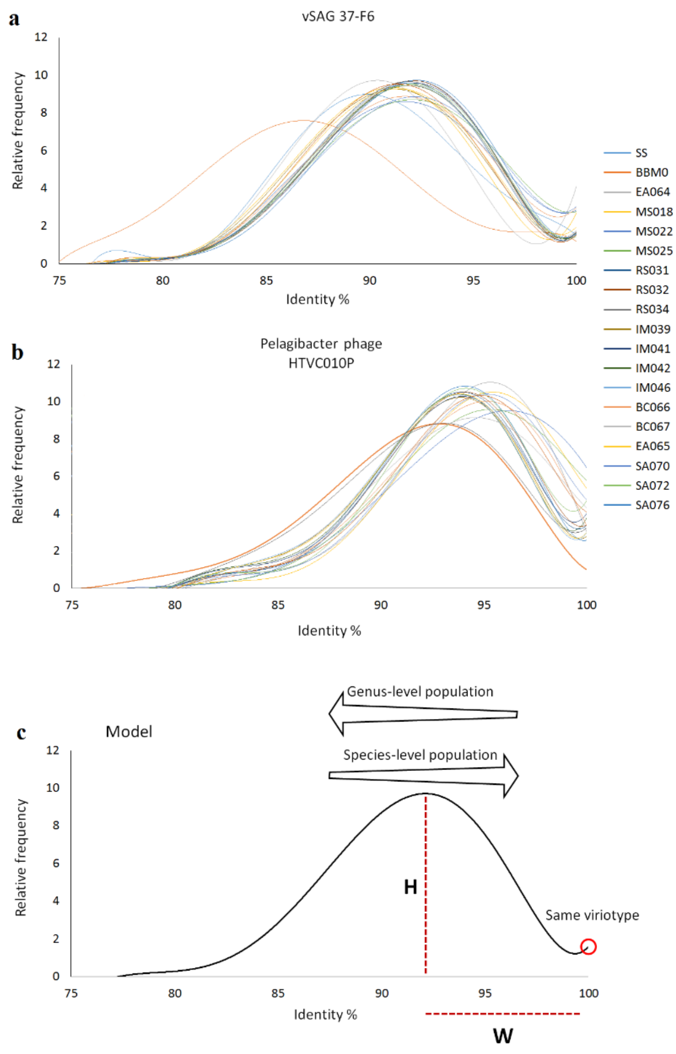

Supplementary Figure 10: Specific-species pattern of viral population of reference viruses in marine environments. (a) Viral population structure of the virus 37-F6 and **(b)** the reference abundant *Pelagibacter* phage in over twenty viromes spanning nearly all oceanic regions. List of abbreviations of viromes as in Fig. 2. Appended numbers refer to the *Tara* metavirome sample nomenclature previously used[7]. Notice that the structure of viral population of virus 37-F6 from the same sampling point (Blanes, Mediterranean Sea) is slightly different than the rest of oceanic regions and based on our proposed model depicted in panel **C** and supplementary text, it is likely more (micro-)diverse in the sampling point than in other regions. **(c)** Proposed model of viral population structure based on metagenomics recruitment inspired by that previously described for prokaryotes[8]. Notice that in contrast to prokaryotes, a genetic discontinuity is not observed between 90-95% of identity but is rather a continuous line with a clear peak precisely in that identity range. None of vSAGs, virus isolates, fosmids and viral contigs recruited reads below 75% identity. Furthermore, a secondary peak observed in prokaryotes at the level of <90% identity is not observed either. Red arrows and dots

depict the biological meaning of recruited viromic reads. H, height of the curve. W, half of the width of the curve.

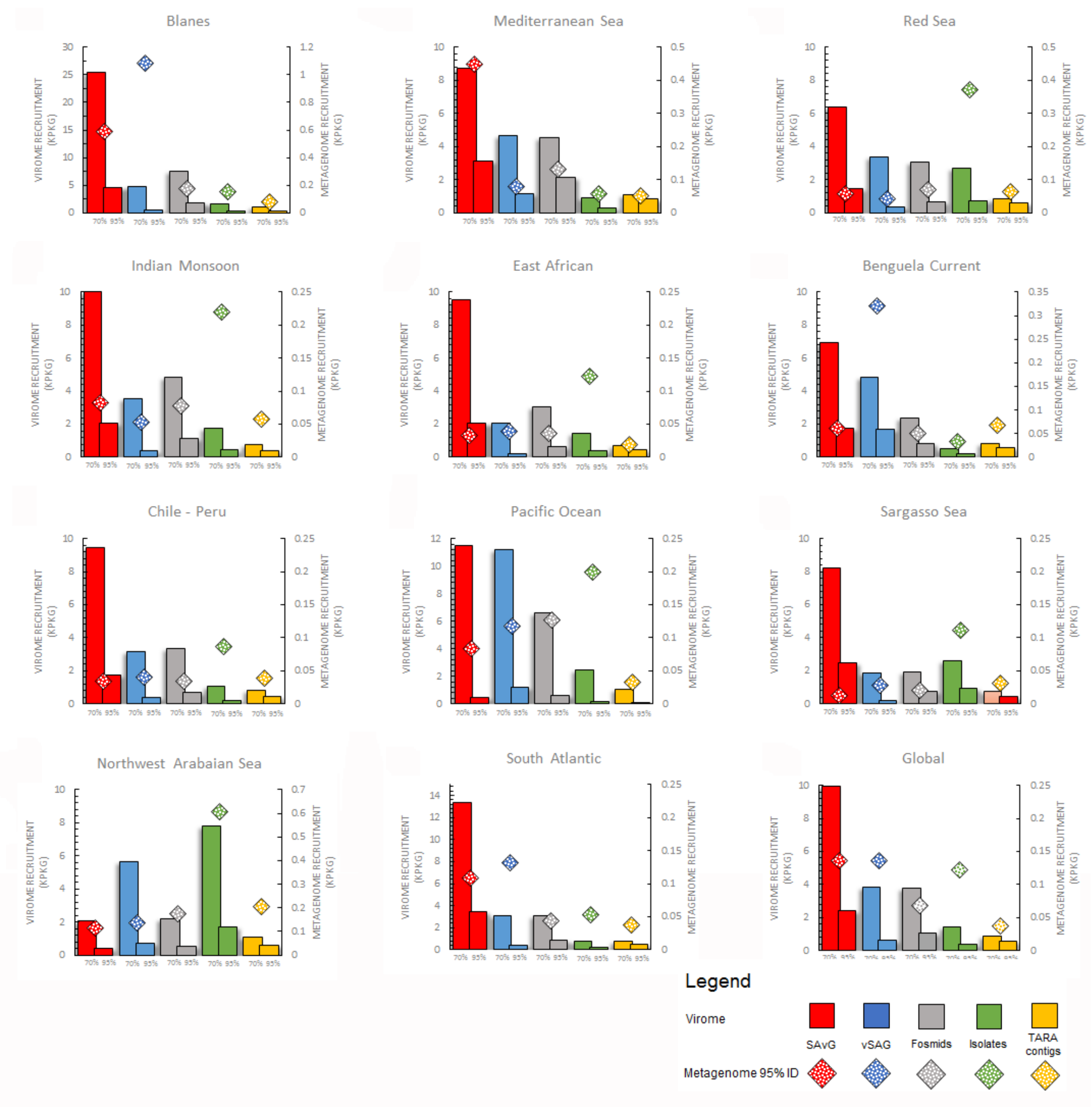

Supplementary Fig. 11. Abundance of viral genome datasets in the different analyzed regions. Virome recruitment (in columns) with different identity thresholds (≥70 and ≥95%). Microbial metagenomic recruitment rate (diamonds) results with an identity threshold of ≥95%. The vSAG dataset showed the highest recruitment rate expressed in recruited kb per kb viral genome per Gb of virome (KPKG) in most of the analyzed viromes, but no significant differences in the microbial metagenomics recruitment were observed among the viral genome datasets.

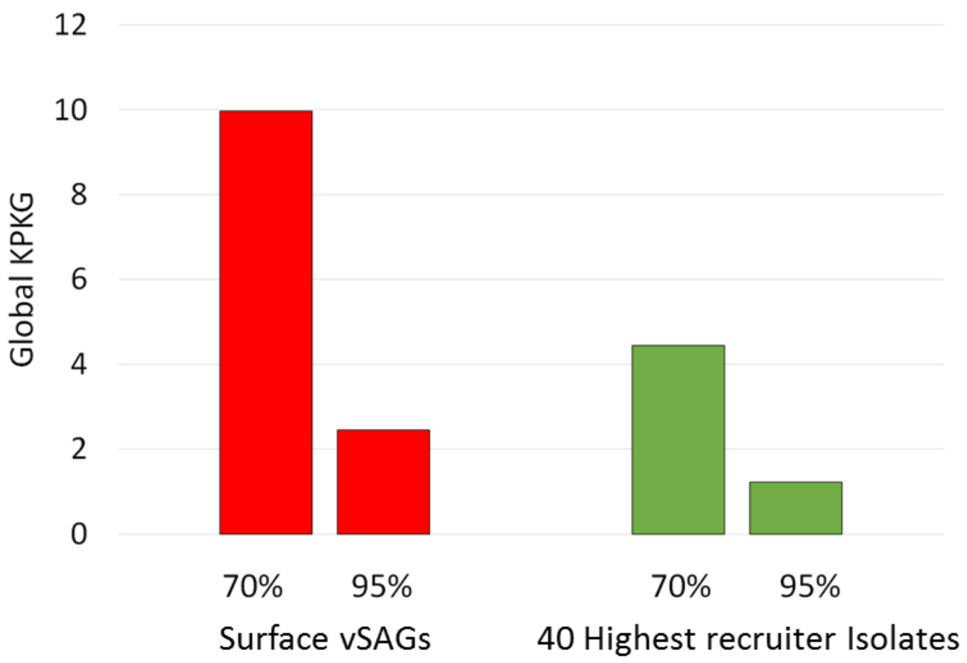

Supplementary Figure 12: Virome recruitment rate of vSAGs compared to the 40 most abundant virus isolates at the global scale. We used two identity thresholds (≥70 and ≥95%**).** In this analysis, we biased in purpose the comparison by considering only those 40 virus isolates with the highest recruitment rate in the surface viriosphere. Even in that scenario, the relative recruitment rate of vSAG was higher.

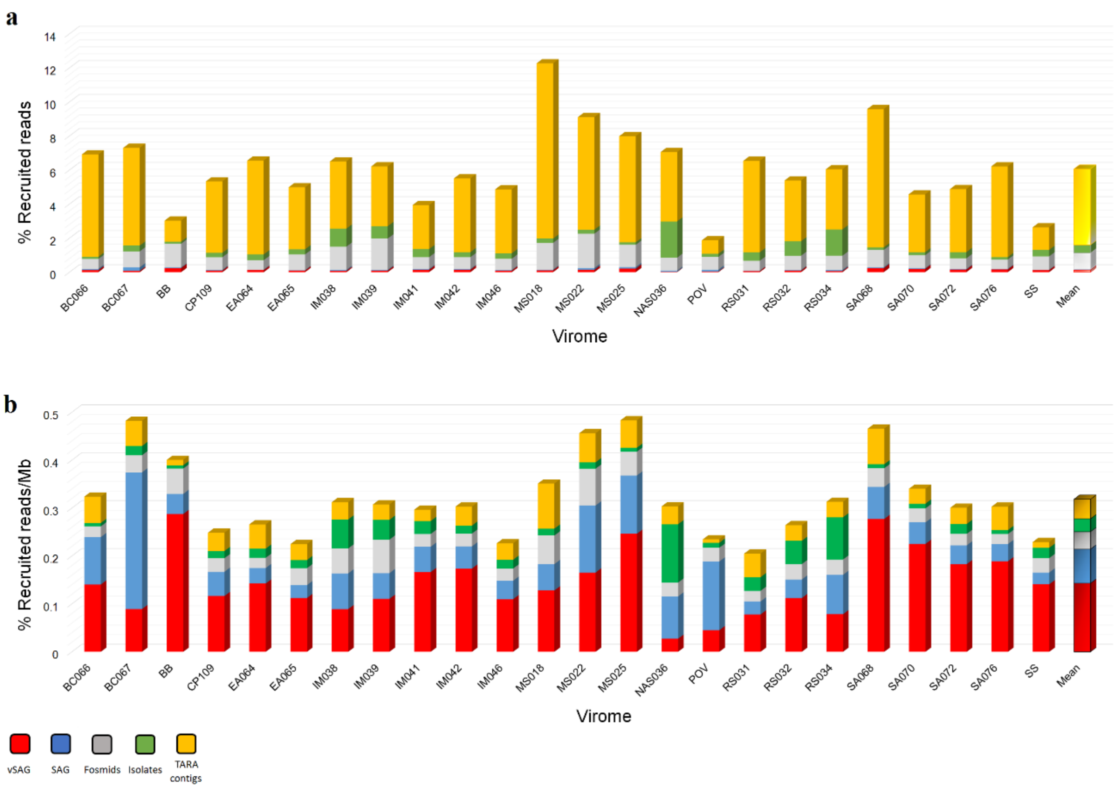

Supplementary Figure 13: Reads recruited for each viral genomic dataset (≥95% cut-off identity). **(a)** Non-normalized viromic recruitment results. **(b)** Normalized viromic recruitment results considering the size of the viral genomic dataset.

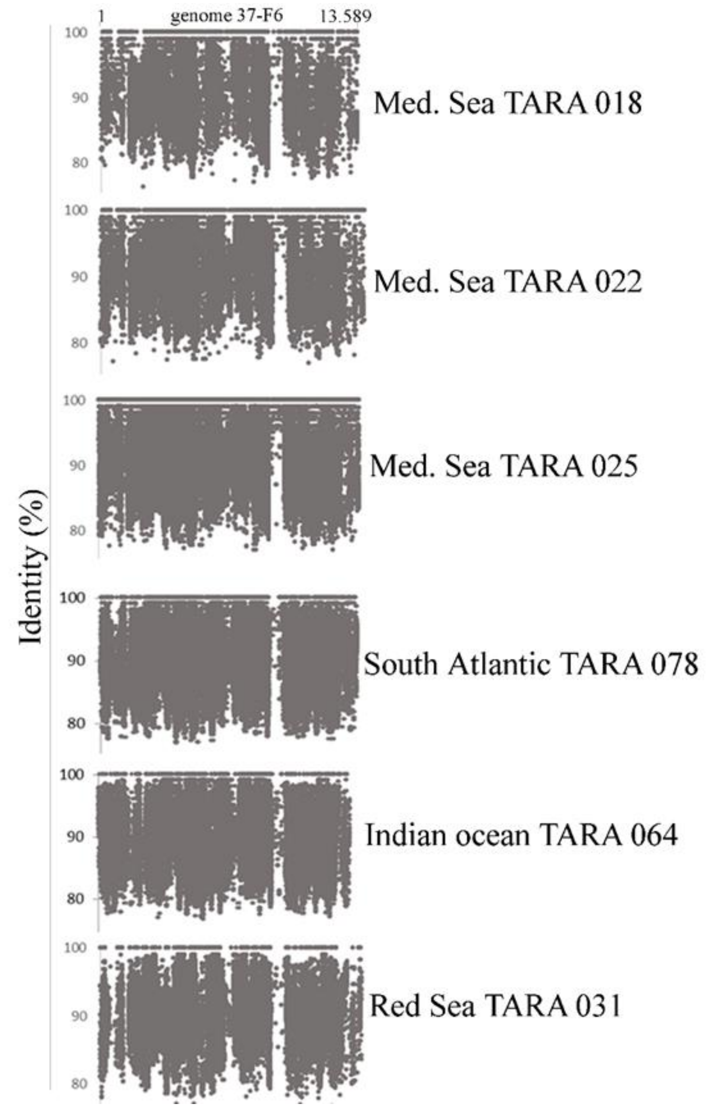

Supplementary Fig. 14. Virome fragment recruitment of the vSAG 37-F6. Virome fragment recruitment in the Indian and the South Atlantic oceans and the Red Sea from *Tara* expedition samples collected several thousand kilometers away from the sampling point of the vSAG 37-F6 (NW Mediterranean, Blanes Bay Microbial Observatory). Note that the genomic island of viruses is almost fully covered in *Tara* Mediterranean viromes from the Western Mediterranean Sea, geographically near to Blanes Bay Microbial Observatory.

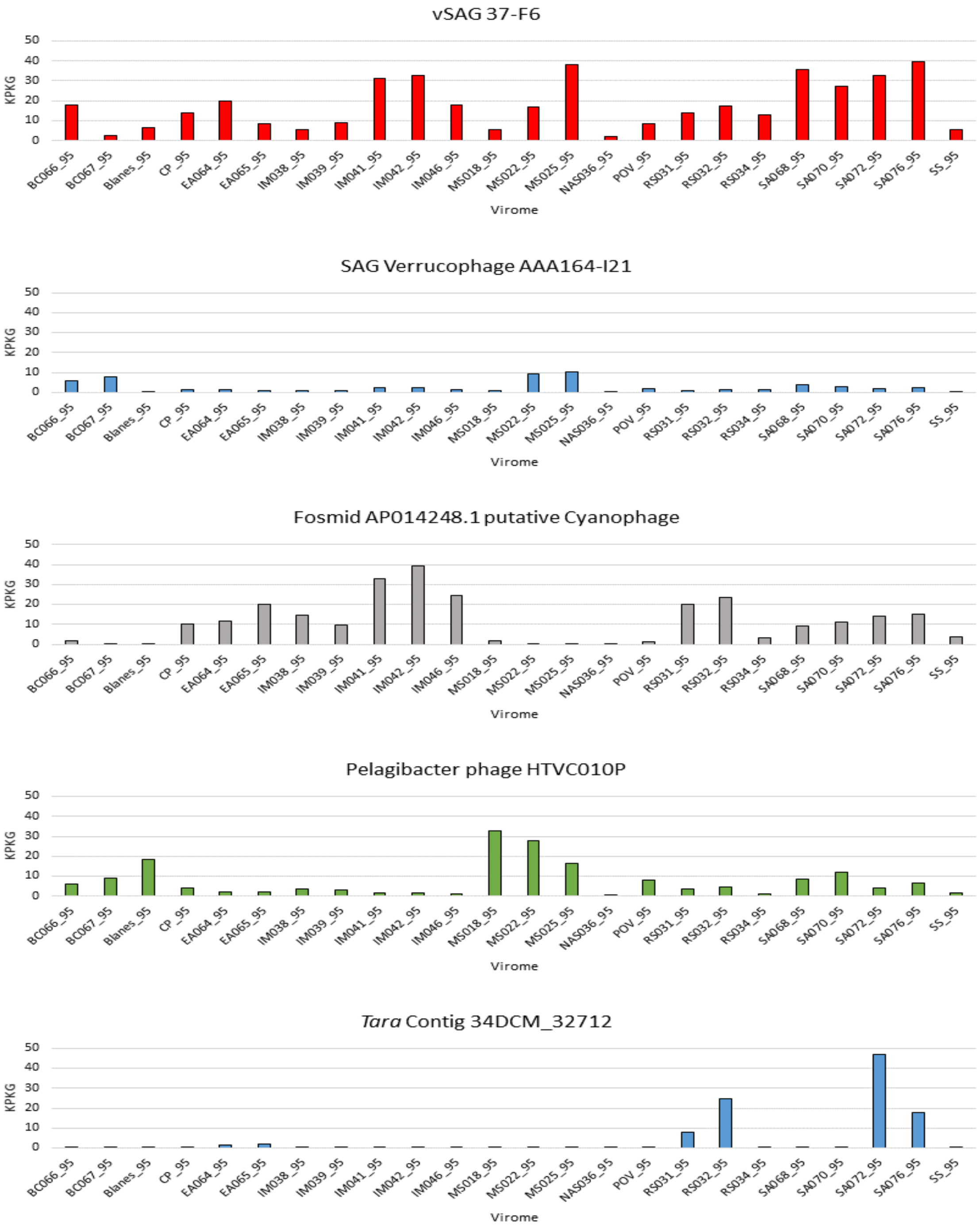

Supplementary Figure 15: Abundance distribution of the most abundant marine viruses. The abundance of the most abundant surface dsDNA viruses for each virus genome datasets according to the procedure for genome recovering (single-virus genomics (37-F6), viruses from single bacterial cells[9] (Verrucophage AAA164-I21), virus cloned in fosmids[10] (AP014248. putative Cyanophage), virus isolates (Pelagibacter phage HTVC010P) and viromics from *Tara* Oceans dataset[11,7] (34DCM_32712), in all viromes. Fragment recruitment data was used to stimate the overall abundance for each region. Abundance is represented in KPKG (as in Fig. 2).

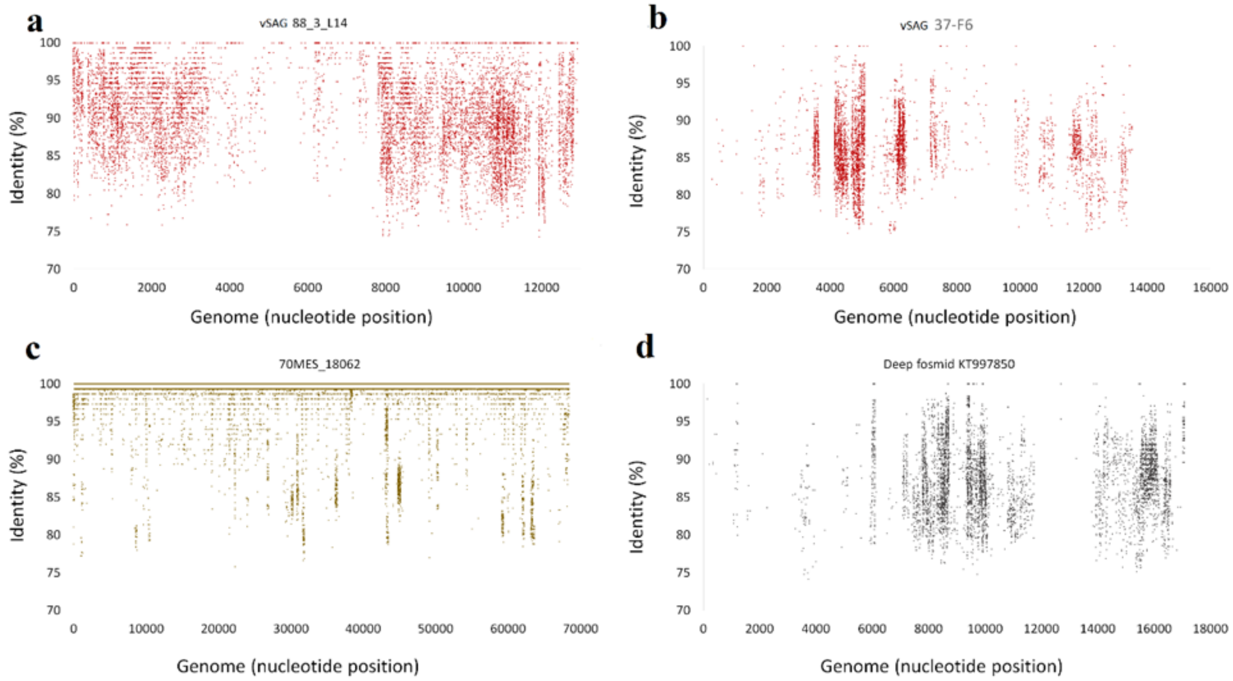

Supplementary Fig. 16. Deep viromic fragment recruitment. Fragment virome recruitment plots from the North Atlantic bathypelagic region (4000 m depth; sample MSP131 from the *Malaspina* Expedition[11]). (**a-d**) Recruitment of the deep vSAG 88-3-L14 was compared with the abundant surface vSAG 37-F6 and those most abundant genome fragments recovered by viromics and cloning in fosmids: *Tara* contig 70_MES_18062 and viral fosmid KT997850[5].

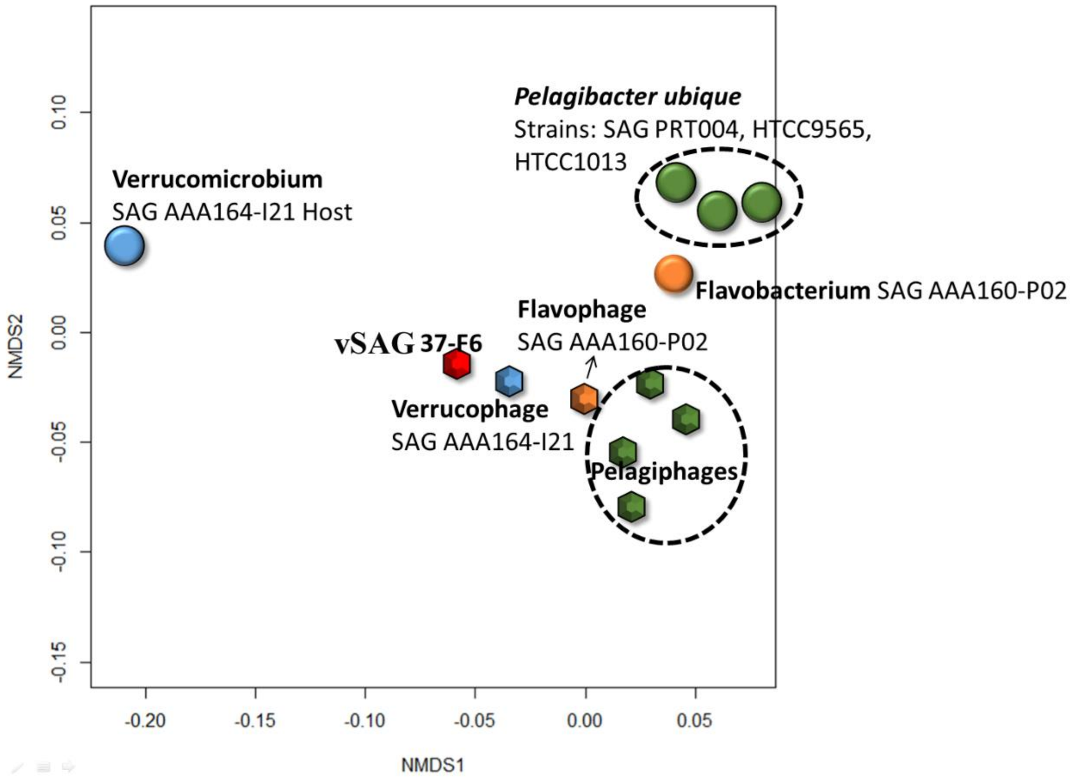

Supplementary Figure 17: Tentative assignation of viruses to hosts according to tetranucleotide frequency signatures. Non-metric MDA of tetranucleotide frequency show the degree of similarity between the different phages and their host with the vSAG 37-F6. Tetranucleotide frequency were calculated with the publicly available bioinformatics tool at the following link: http://mobyle.pasteur.fr/cgi-bin/portal.py#forms::compseq

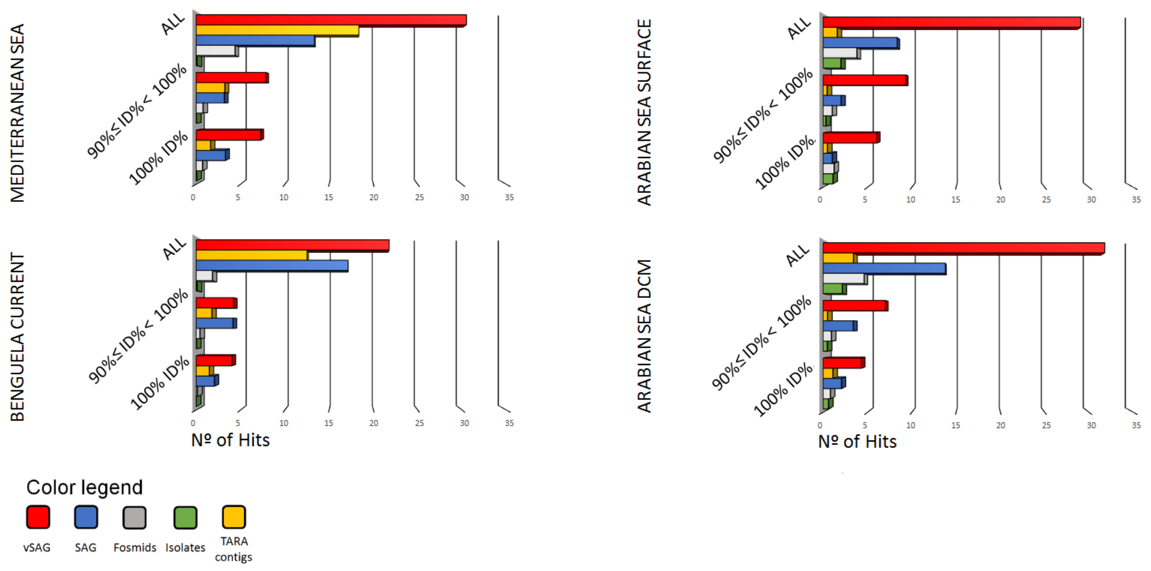

Supplementary Fig. 18. Peptide recruitment for each viral genomic dataset using predicted peptide sequences obtained from *Tara* expedition[12]. Different cut-off identities were used (no cut off, ≥90 but <100%, and 100%). In all four metaproteomes, vSAGs are the most peptide recruiters.

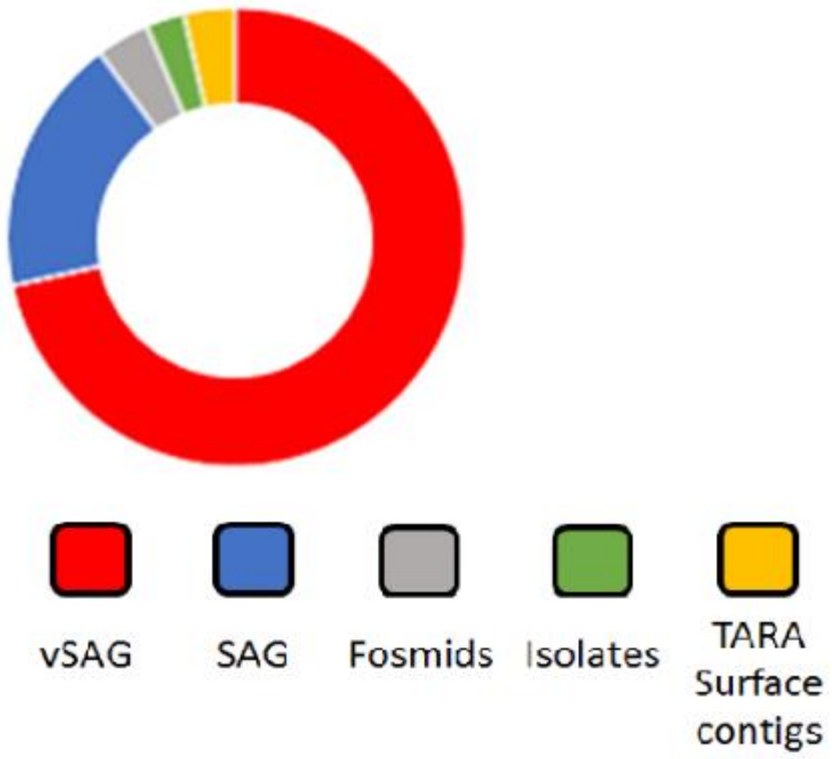

Supplementary Fig. 19. Peptide recruitment (100% identity) for each viral genomic dataset using the predicted peptide sequences obtained in the Oregon Coast[13].

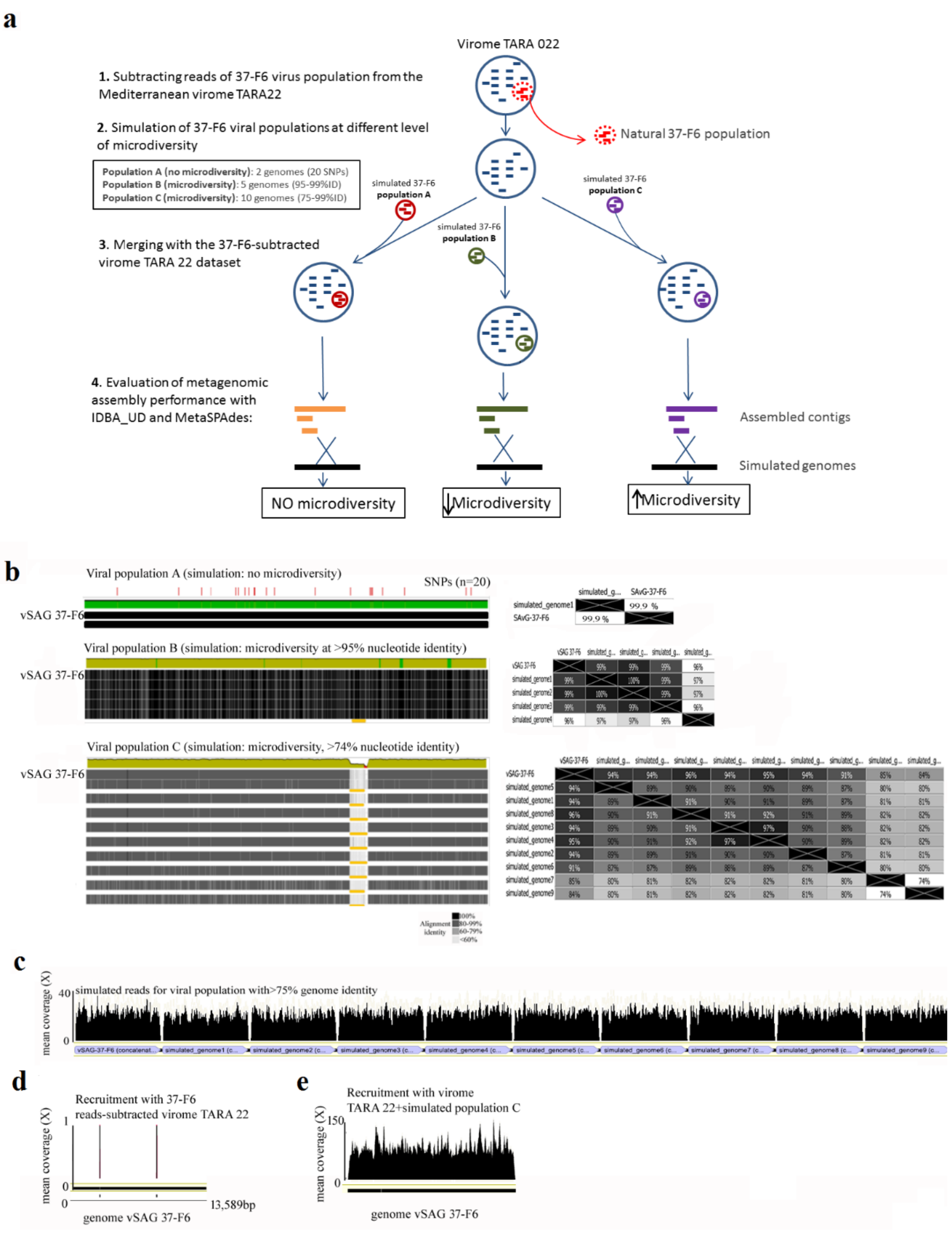

Supplementary Fig. 20. Employed methodology to assess the effect of (micro)-diversity on the metagenomics viral assembly (a) Schematic diagram illustrating the employed methodology to evaluate the metagenomic assembly performance of assemblers to reconstruct the viral genome from populations with different degrees of diversity and microdiversity within a natural virome. First, raw reads from vSAG 37-F6 were removed from *Tara* virome MS022 (see panel d). Then, simulated reads from the

three populations with different level of microdiversity were introduced within *Tara* virome MS022 (see panels b and c). **(b)** Three viral populations of vSAG 37-F6 were simulated (see Methods for details). Population A: no microdiversity; population B: low microdiverse; and population C: medium-high microdiverse. **(c)** For each population**,** Illumina raw reads were generated (see Methods) to simulate the viral populations. Read mapping of those simulated reads against the reference simulated genomes of vSAG at different microdiversity degrees confirmed that all genomes had at least a genome coverage of 40X. For convenience, only the simulation and mapping of reads is shown for the population C. **(d)** Reads corresponding for the vSAG population 37-F6 were removed from virome *Tara* MS022. **(e)** Mapping of simulated virome *Tara* MS022 with the introduced population C of vSAG 37-F6 confirmed that raw reads mapped with high coverage against the reference simulated genomes. For convenience, only data is shown for the population C.

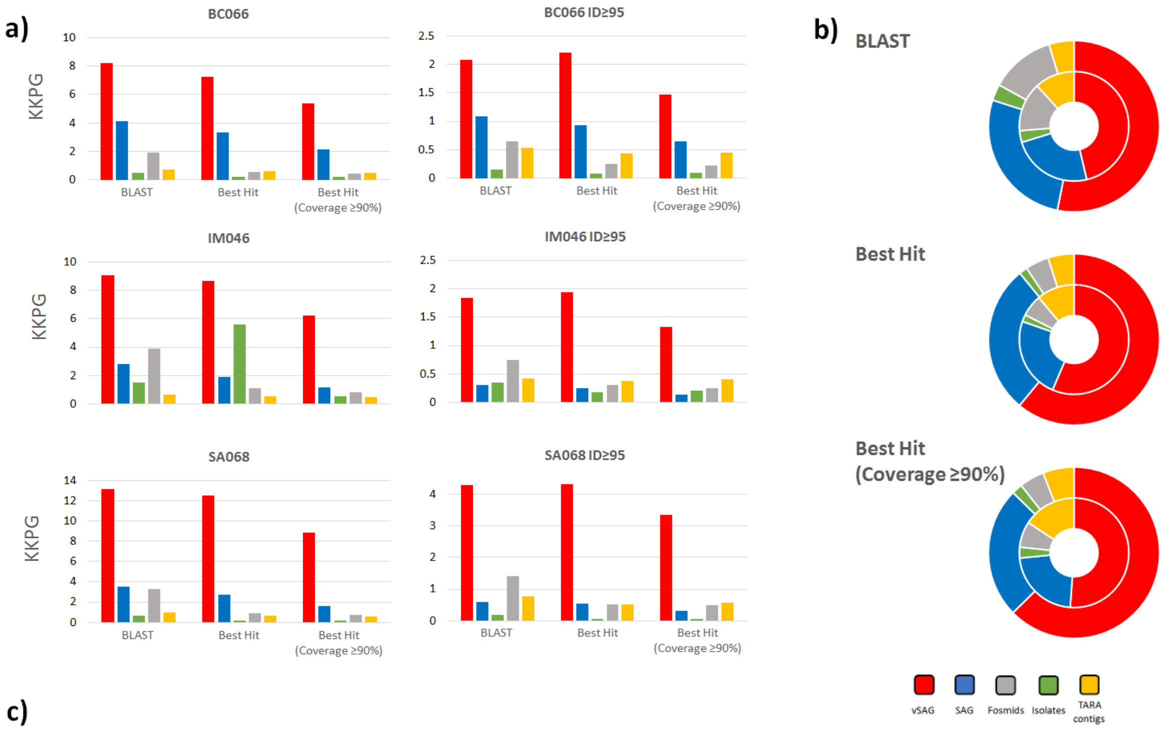

c)

	BC066			BC066 ID ≥ 95%			IM046			IM046 ID ≥ 95%			SA068			SA068 ID ≥ 95%		
	BLAST	Best Hit	Best Hit Cov ≥ 90%	BLAST	Best Hit	Best Hit Cov ≥ 90%	BLAST	Best Hit	Best Hit Cov ≥ 90%	BLAST	Best Hit	Best Hit Cov ≥ 90%	BLAST	Best Hit	Best Hit Cov ≥ 90%	BLAST	Best Hit	Best Hit Cov ≥ 90%
vSAG	8.223	7.221	5.39	2.085	2.206	1.473	9.086	8.656	6.243	1.833	1.941	1.331	13.166	12.519	8.839	4.297	4.322	3.362
SAG	4.148	3.323	2.127	1.08	0.935	0.642	2.824	1.908	1.139	0.305	0.245	0.128	3.499	2.729	1.627	0.602	0.543	0.312
Isolates	0.475	0.186	0.174	0.155	0.081	0.093	1.518	5.571	0.543	0.343	0.183	0.202	0.681	0.195	0.158	0.177	0.062	0.059
Fosmids	1.894	0.525	0.405	0.653	0.251	0.219	3.862	1.081	0.822	0.751	0.304	0.25	3.302	0.891	0.736	1.414	0.507	0.479
Tara Contigs	0.733	0.581	0.506	0.531	0.435	0.453	0.663	0.55	0.469	0.413	0.375	0.403	0.986	0.664	0.598	0.781	0.516	0.564

Supplementary Fig. 21. Comparison of different algorithms for matagenomic fragment recruitment. We compared the method that we used in our metagenomic fragment recruitment (**Fig. 2**) previously used by other authors[10] with the reciprocal-best hit fragment recruitment employed in the study of Pelagibacter phages[14]. Best-hit fragment recruitment was carried out with the Enveomics bioinformatic package (https://peerj.com/preprints/1900/) as described. Two fragment recruitment variants were also tested: without query coverage filtering and applying 90% of query coverage cut-off. **a)** Fragment recruitment with three different viromes are shown, Benguela Current (BC066), Indian Monsoon (IM046), and Southern Atlantic (SA068), using a 70% and 95% Identity cut-off. **b)** Relative fragment recruitment with Benguela Current virome (BC066). **c)** Data of the three recruitments. Overall, data indicate that no differences were observed among recruiter strategies.

Supplementary Tables

Supplementary Table 1. Sequencing results and assembly for the marine vSAGs

vSAG	Sample$^{\alpha}$	Treatment$^{\beta}$	Contigs	GC%	Sequence Length (bp)
**17-C23**	1	A	17-C23-contig1	35.10	78,637
			17-C23-contig2	39.00	7,850
**17-D16**	1	A	17-D16	30.30	12,025
**17-D19**	1	A	17-D19-contig1	34.10	7,108
			17-D19-contig20	35.00	14,151
**17-E11***	1	A	17-E11	36.30	6,957
**17-E15**	1	A	17-E15	34.60	33,035
**17-F13**	1	A	17-F13	38.40	33,869
**17-F19**	1	A	17-F19-contig1	36.30	15,706
			17-F19-contig2	35.80	2,525
			17-F19-contig3	35.90	2,236
**17-G23**	1	A	17-G23-contig1	32.40	7,276
			17-G23-contig2	32.60	11,351
**37-D17**	2	B	37-D17	34.20	8,248
**37-F6***	2	B	37-F6	38.20	13,589
**37-F16**	2	B	37-F16	30.90	58,722
**37-G23**	2	B	37-G23	36.00	11,565
**37-H5**	2	B	37-H5-contig1	37.60	25,858
			37-H5-contig2	39.50	18,835
**37-I21***	2	B	37-I21	36.10	31,959
**37-J6**	2	B	37-J6-contig1	33.70	23,751
			37-J6-contig2	32.80	6,530
**37-K7**	2	B	37-K7-contig1	35.10	2,871
			37-K7-contig2	35.90	10,586
			37-K7-contig3	37.80	8,957
			37-K7-contig4	35.00	8,189
**37-K11**	2	B	37-K11	34.50	13,098
**37-L15***	2	B	37-L15-contig1	31.70	16,494
			37-L15-contig2	34.00	13,846
			37-L15-contig3	30.20	2,160
**37-M8**	2	B	37-M8	36.50	10,162
**37-M19**	2	B	37-M19	35.20	20,541
**37-P14**	2	B	37-P14	35.90	7,161
**40-A23**	2	B	40-A23	36.90	4,388
**40-B17**	2	B	40-B17	33.50	5,502
**40-B18**	2	B	40-B18	38.20	20,323
**40-D19**	2	B	40-D19	33.50	23,628
**40-H15**	2	B	40-H15	33.70	7,577
**40-J13**	2	B	40-J13	44.50	4,380

**40-L14**	2	B	40-L14	37.70	8,282
**40-P19**	2	B	40-P19	31.20	6,640
**41-A4**	2	C	41-A4-contig1	36.80	13,834
			41-A4-contig2	37.80	18,697
**41-D7***	2	C	41-D7-contig1	32.60	24,030
			41-D7-contig2	34.00	14,432
**41-D13**	2	C	41-D13	32.80	6,045
**41-H4**	2	C	41-H4-contig1	28.50	36,279
			41-H4-contig2	29.60	17,198
			41-H4-contig3	29.20	10,721
**41-H16**	2	C	41-H16	39.20	11,145
**41-H17**	2	C	41-H17	35.50	6,664
**41-I9**	2	C	41-I9	31.20	4,913
**41-I14**	2	C	41-I14	36.20	28,554
**41-I16**	2	C	41-I16	34.10	7,028
**41-I18**	2	C	41-I18-contig1	36.30	8,360
			41-I18-contig2	34.20	8,389
**41-O11**	2	C	41-O11	37.20	14,512
**80-3-I13**	3	B	80-3-I13	36.60	22,966
**30-E13**	4	E	30-E13	44.50	37,588
**30-J17**	4	E	30-J17	32.90	17,011
**88-3-L14**	5	E	88-3-L14	37.20	12,924

*Two different sequencing were done, using Nextera and True Seq;
$^{\alpha}$Sample: 1=Mediterranean Sea, Barceloneta Beach; 2=Mediterranean Sea, Blanes Bay Microbial Observatory; 3=Mediterranean Sea DCM; 4=North Atlantic Ocean, Malaspina expedition sample 134; 5=North Atlantic Ocean, Malaspina expedition sample 131Treatment$^{\beta}$: A=fixed sample+liquid N_2 and KOH (pH14) shock; B=unfixed sample+liquid N_2 and KOH (pH=14) shock; C=unfixed sample+KOH (pH=14) shock.; E=cryopreserved in GlyTE+treatment B

Supplementary Table 2: Relatedness of vSAGs with the Global Oceanic Viral Clusters [11] and tentative taxonomy prediction based on gene-content network analysis (see methods for details)

Sequence	vSAG	Closest VC (this study)	VC Size	GOV VC (Roux et al, 2016)	No. of GOV	No. of vSAGs	References	Order*	Family*	Genus*
17-C23-contig1	17-C23	VC_0078	34	VC_0434	20	2	12	Caudovirales (12)	Siphoviridae (12)	T5 like virus (8)
17-C23-contig2	17-C23									
17-D16	17-D16	VC_0234	8	VC_0446	7	1	0			
17-D19-contig1	17-D19	VC_0003	626	VC_0006	616	6	0			
17-D19-contig2	17-D19	VC_0003	626	VC_0006	616	6	0			
17-E11	17-E11	VC_0005	467	VC_0008	461	3	0			
17-E15	17-E15	VC_0408	4	VC_1116	3	1	0			
17-F13	17-F13	VC_0156	14	VC_0303	13	1	0			
17-F19-contig1	17-F19	VC_0013	205	VC_0019	199	5	1	Caudovirales (1)	Podoviridae (1)	
17-F19-contig2	17-F19	VC_0013	205	VC_0019	199	5	1			
17-F19-contig3	17-F19									
17-G23-contig1	17-G23	VC_0052	58	VC_0095	57	1	0			
17-G23-contig2	17-G23	VC_0158	14	VC_0281	13	1	0			
30-E13	30-E13	VC_0087	30	VC_0165	28	1	1	Caudovirales (1)	Siphoviridae (1)	
30-J17	30-J17	VC_0110	23	VC_0143	22	1	0			
37-D17	37-D17	VC_0002	678	VC_0005	665	7	5	Caudovirales (5)	Podoviridae (5)	
37-F16	37-F16	VC_0014	195	VC_0031	190	2	1	Caudovirales (1)	Myoviridae (1)	
37-F6	37-F6	VC_0005	467	VC_0008	461	3	0			
37-G23	37-G23	VC_0089	29	VC_0176	27	2	0			
37-H5-contig1	37-H5	VC_0013	205	VC_0019	199	5	1	Caudovirales (1)	Podoviridae (1)	
37-H5-contig2	37-H5	VC_0023	136	VC_0019	125	5	1	Caudovirales (1)	Podoviridae (1)	

37-I21	37-I21	VC_0078	34	VC_0434	20	2	12	Caudovirales (12)	Siphoviridae (12)	T5 like virus (8)
37-J6-contig1	37-J6	VC_0002	678	VC_0005	665	7	5	Caudovirales (5)	Podoviridae (5)	
37-J6-contig2	37-J6	VC_0002	678	VC_0005	665	7	5			
37-K11	37-K11	VC_0022	141	VC_0047	138	1	2	Caudovirales (1)	Podoviridae (1)	
37-K7-contig1	37-K7									
37-K7-contig2	37-K7	VC_0033	102	VC_0060	98	3	0			
37-K7-contig3	37-K7	VC_0023	136	VC_0019	125	5	1	Caudovirales (1)	Podoviridae (1)	
37-K7-contig4	37-K7	VC_0033	102	VC_0060	98	3	0			
37-L15-contig1	37-L15	VC_0013	205	VC_0019	199	5	1	Caudovirales (1)	Podoviridae (1)	
37-L15-contig2	37-L15	VC_0068	39	VC_0155	12	1	0			
37-L15-contig3	37-L15									
37-M19	37-M19	VC_0039	83	VC_0054	82	1	0			
37-M8	37-M8	VC_0027	123	VC_0067	80	1	0			
37-P14	37-P14	VC_0023	136	VC_0019	125	5	1	Caudovirales (1)	Podoviridae (1)	
40-A23	40-A23	VC_0003	626	VC_0006	616	6	0			
40-B17	40-B17	VC_0033	102	VC_0060	98	3	0			
40-B18	40-B18	VC_0017	168	VC_0029	167	1	0			
40-D19	40-D19	VC_0012	210	VC_0027	208	1	0			
40-H15	40-H15	VC_0000	1090	VC_0002	970	1	49	Caudovirales (48)	Myoviridae (45)	T4 like virus (18)
40-J13	40-J13	VC_0733	2		1	1	0			
40-L14	40-L14	VC_0003	626	VC_0006	616	6	0			
40-P19	40-P19	VC_0023	136	VC_0019	125	5	1	Caudovirales (1)	Podoviridae (1)	
41-A4-contig1	41-A4	VC_0005	467	VC_0008	461	3	0			
41-A4-contig2	41-A4	VC_0216	9	VC_0384	8	1	0			
41-D13	41-D13	VC_0701	2		1	1	0			

41-D7-contig1	41-D7	VC_0002	678	VC_0005	665	7	5	Caudovirales (5)	Podoviridae (5)
41-D7-contig2	41-D7	VC_0089	29	VC_0176	27	2	0		
41-H16	41-H16	VC_0023	136	VC_0019	125	5	1	Caudovirales (1)	Podoviridae (1)
41-H17	41-H17	VC_0003	626	VC_0006	616	6	0		
41-H4-contig1	41-H4	VC_0016	193	VC_0033	191	1	0		
41-H4-contig2	41-H4	VC_0014	195	VC_0031	190	2	1	Caudovirales (1)	Myoviridae (1)
41-H4-contig3	41-H4	VC_0001	751	VC_0003	750	1	0		
41-I14	41-I14	VC_0088	30	VC_0171	29	1	0		
41-I16	41-I16	VC_0002	678	VC_0005	665	7	5	Caudovirales (5)	Podoviridae (5)
41-I18-contig1	41-I18	VC_0013	205	VC_0019	199	5	1	Caudovirales (1)	Podoviridae (1)
41-I18-contig2	41-I18	VC_0267	7	VC_0525	6	1	0		
41-I9	41-I9	VC_0002	678	VC_0005	665	7	5	Caudovirales (5)	Podoviridae (5)
41-O11	41-O11	VC_0045	68	VC_0090	67	1	0		
80-3-I13	80-3-I13	VC_0002	678	VC_0005	665	7	5	Caudovirales (5)	Podoviridae (5)
88-3-L14	88-3-L14	VC_0003	626	VC_0006	616	6	0		

*Taxonomic affiliation of vSAG at the level of Family or Order is tentative and has to be taken very cautious since there is no experimental proof

Supplementary Table 3. Comparison at the population level of the single-viruses with viral clusters obtained in the Global Ocean Virome (GOV) dataset[11]

vSAG	Putative assignment[α]	VC_2	VC_3	VC_5	VC_6	VC_8	VC_9
17-D16	VC6				**52**		
17-D19	VC6				**184**	11	
17-E11	VC8				35	**236**	
37-D17	VC5			345			
37-J6	VC5			**1041**			
37-K11	VC5	9		**121**	4	4	19
37-F6	VC8				59	**413**	
40-A23	VC6				**10**		
40-B18	VC5			**15**			
40-H15	VC2	**10**					
40_L14	VC6				**89**		
41-A4	VC8				89	**566**	
41-D7	VC5		37	**1162**			
41-H17	VC6				**11**	1	
41-H4	VC3	2	**40**	8			
41-I14	VC2	171	1				
41-I16	VC5			**236**			
41-I9	VC5			**146**			
41-O11	VC2	92	9	1			

[α]Assignment was done based on genomic comparison by BLASTn against all bins and viral contigs in the GOV dataset. Only hits with GOV dataset with the following criteria were considered for assignment: bitscore threshold hit>100, sequence alignment length>500 bp, >10 hits spanning the genome and ≥80% of hits accumulated within the same VC. Alignment mean identity of hits with viral contigs/bins of VC was ≈70%. vSAGs not listed in the table showed an uncertain assignment

Supplementary Table 4. Pairwise BLASTp comparison of vSAG with the closest virus in the global marine viral clusters (VCs) based on protein-sharing network analysis.

vSAG	vSAG (contig)* - Closest virus in VC	No. of shared proteins	No. of total vSAG genes	%Pairwise	Putative new species (NS) or new genera (NG)[β]
**vSAG-17-C23**	GOV_bin_5106_contig-100_0	25	116	48.60	NS
**vSAG-17-D16**	GOV_bin_1735_contig-100_0	15	19	62.56	NS
**vSAG-17-D19**	Contig 1- Tp1_123_SUR_0-0d2_scaffold29973_1<br>Contig 2- Tp1_123_DCM_0-0d2_scaffold46460_2	8<br>11	11<br>16	55.00<br>56.45	NS
**vSAG-17-E11**	GOV_bin_2164_contig-100_0	3	11	56.00	NS
**vSAG-17-E15**	GOV_bin_4005_contig-100_0	10	35	38.70	NS
**vSAG-17-F13**	GOV_bin_870_contig-100_1	11	14	56.69	NS
**vSAG-17-F19**	Contig 1-Tp1_30_DCM_0-0d2_scaffold60669_1<br>Contig 2-GOV_bin_534_contig-100_2<br>Contig 3-*No Closest*	16<br>5<br>0	20<br>5<br>3	99.73<br>56.96<br>---	NS
**vSAG-17-G23**	Contig 1-Tp1_23_DCM_0-0d2_scaffold128056_1<br>Contig 2-Tp1_23_DCM_0-0d2_scaffold112175_1	7<br>11	10<br>13	59.93<br>52.85	NS
**vSAG-30-E13**	GOV_bin_636_contig-100_5	6	31	41.50	NS
**vSAG-30-J17**	GOV_bin_8033_contig-100_1	7	11	56.00	NS
**vSAG-37-D17**	GOV_bin_3340_contig-100_6	8	11	67.36	NG
**vSAG-37-F16**	vSAG-41-H4-contig2	15	54	69.67	NS
**vSAG-37-F6**	SAG AAA164-I21-contig 5	18	24	65.16	NS
**vSAG-37-G23**	GOV_bin_4091_contig-100_8	14	18	60.30	NS
**vSAG-37-H5**	Contig 1-GOV_bin_1874_contig-100_1<br>Contig 2-Tp1_30_DCM_0-0d2_scaffold21665_1	19<br>16	36<br>27	56.07<br>61.41	NS
**vSAG-37-I21**	Tp1_82_SUR_0-0d2_scaffold12183_1	18	35	47.69	NS
**vSAG-37-J6**	Contig 1-Tp1_36_DCM_0-0d2_scaffold99746_1<br>Contig 2-GOV_bin_3099_contig-100_0	18<br>6	34<br>6	69.97<br>37.58	NS
**vSAG-37-K11**	Tp1_102_SUR_0-0d2_scaffold55818_1	5	17	72.44	NS
**vSAG-37-K7**	Contig 1- *No Closest*<br>Contig 2- GOV_bin_5817_contig-100_0<br>Contig 3- GOV_bin_4362_contig-100_0<br>Contig 4- Tp1_32_SUR_0-0d2_scaffold63617_1	0<br>5<br>8<br>5	11<br>6<br>11<br>12	---<br>42.08<br>64.80<br>61.26	NG
**vSAG-37-L15**	Contig 1-GOV_bin_3005_contig-100_2<br>Contig 2-Uncultured_Mediterranean_phage_uvMED_AP014493<br>Contig 3-*No Closest*	5<br>10<br>0	37<br>20<br>2	52.64<br>55.71<br>---	NS
**vSAG-37-M19**	GOV_bin_2674_contig-100_1	27	37	79.50	NS

**vSAG-37-M8**	Tp1_100_DCM_0-0d2_scaffold6111_1	14	22	74.10	NS
**vSAG-37-P14**	Tp1_123_DCM_0-0d2_scaffold44431_1	6	6	46.58	NG
**vSAG-40-A23**	Tp1_111_DCM_0-0d2_scaffold17799_1	8	10	70.46	NS
**vSAG-40-B17**	Tp1_111_DCM_0-0d2_scaffold53353_1	13	15	75.85	NG
**vSAG-40-B18**	Tp1_31_SUR_0-0d2_scaffold205369_1	21	29	61.56	NS
**vSAG-40-D19**	GOV_bin_4866_contig-100_1	14	36	53.90	NS
**vSAG-40-H15**	Uncultured_Mediterranean_phage_uvMED_AP014348	7	8	62.63	NS
**vSAG-40-J13**	GOV_bin_8324_contig-100_4	5	10	88.68	NS
**vSAG-40-L14**	vSAG-17-D19-contig1	8	12	53.43	NS
**vSAG-40-P19**	Tp1_124_SUR_0-0d2_scaffold12109_4	4	18	65.23	NS
**vSAG-41-A4**	Contig 1-GOV_bin_4626_contig-100_1<br>Contig 2- GOV_bin_2910_contig-100_1	22<br>17	34<br>29	55.80<br>49.59	NS
**vSAG-41-D13**	GOV_bin_6709_contig-100_0	7	14	60.84	NG
**vSAG-41-D7**	Contig 1-GOV_bin_2729_contig-100_2<br>Contig 2- GOV_bin_7344_contig-100_5	14<br>10	31<br>20	56.44<br>67.00	NS
**vSAG-41-H16**	Tp1_125_DCM_0-0d2_scaffold6988_1	7	7	58.06	NS
**vSAG-41-H17**	Uncultured_Mediterranean_phage_uvMED_AP014380	13	19	65.05	NS
**vSAG-41-H4**	Contig 1-GOV_bin_3401_contig-100_0<br>Contig 2-vSAG-37-F16<br>Contig 3- GOV_bin_5740_contig-100_6	20<br>15<br>9	39<br>20<br>14	56.89<br>68.81<br>55.29	NS
**vSAG-41-I14**	Tp1_102_DCM_0-0d2_scaffold2867_3	24	47	70.58	NS
**vSAG-41-I16**	GOV_bin_3340_contig-100_6	5	9	55.43	NS
**vSAG-41-I18**	Contig 1-Tp1_22_SUR_0-0d2_scaffold30721_1<br>Contig 2- GOV_bin_3845_contig-100_3	5<br>5	10<br>10	45.92<br>78.06	NG
**vSAG-41-I9**	Tp1_66_SUR_0-0d2_scaffold28495_4	4	4	68.88	NS
**vSAG-41-O11**	GOV_bin_4674_contig-100_0	14	27	58.72	NS
**vSAG-80-3-I13**	GOV_bin_2729_contig-100_2	16	22	58.86	NS
**vSAG-88-3-L14**	Tp1_25_DCM_0-0d2_scaffold2249_3	5	15	54.28	NG
**MEAN**		**11.29**	**20.73**	**60.38**	

*In case two or more genome fragments (viral contig) were obtained from the vSAG, the closest viral genome in database is indicate

$^{\beta}$ Although application of viral taxonomy criteria to define viral species and genera remains complicated to uncultured viruses, in this study we have used the following criteria based on a previous study[4]. New genera are defined when the vSAGs presented weaker connections with closest viral relatives within the global marine viral network, as previously described[4]. New viral species are defined when ≤95% of nucleotide identity was obtained with the closest viral relative.

Supplementary Table 5. Ranking of the first most recruiter viruses at different cut-off identities (70 and 95%) in different oceanic regions[7,11,15,16] for each viral datasets (single-viruses, fosmids[10], virus isolates (Supplementary Table 9), viruses from microbial single amplified genomes (SAGs) cells[9], viral genomes reconstructed by viromics from *Tara* Ocean Viromes (TOV)[7] and Global Ocean Viromes (GOV)[11])

**Viral genome dataset[±]**	**vSAG 37-F6[£]**		**vSAG**		**SAGs**		**Fosmids**		**Isolates**		**TOV**		**GOV**	
**ID %**	**70**	**95**	**70**	**95**	**70**	**95**	**70**	**95**	**70**	**95**	**70**	**95**	**70**	**95**
**VIROME***														
**SS**	**1**	55	**1**	14	67	652	5	7	8	6	7	**1**	2	3
**POV**	3	15	3	15	8	14	**1**	**1**	20	18	10	2	11	26
**BBMO**	24	99	**1**	7	134	268	3	**1**	18	18	122	24	2	12
**CP109**	6	18	6	17	76	172	10	24	205	140	32	2	**1**	**1**
**MS018**	66	369	66	91	145	398	**1**	**1**	14	59	25	9	113	74
**MS022**	7	62	7	58	55	358	**1**	4	16	29	11	3	10	**1**
**MS025**	**1**	2	**1**	2	10	115	5	**1**	32	65	38	14	4	3
**RS031**	**1**	7	**1**	7	61	839	2	4	87	196	9	**1**	5	2
**RS032**	**1**	18	**1**	18	83	823	3	14	49	36	5	2	2	**1**
**RS034**	6	26	6	26	38	115	8	3	**1**	**1**	37	13	2	2
**NAS036**	238	631	238	631	35	128	9	26	2	10	7	3	**1**	**1**
**IM038**	41	215	41	67	45	292	**1**	**1**	16	18	26	6	18	12
**IM039**	39	111	39	63	121	665	**1**	**1**	55	51	72	30	60	34
**IM041**	**1**	2	**1**	2	30	158	3	**1**	130	112	10	8	7	10
**IM042**	**1**	2	**1**	2	38	216	2	**1**	171	96	19	3	9	29
**IM046**	**1**	10	**1**	10	79	597	2	3	161	235	36	5	9	**1**
**EA064**	**1**	2	**1**	**1**	29	551	5	3	16	4	9	6	4	7
**EA065**	8	58	8	29	160	733	**1**	**1**	37	15	67	4	9	43
**BC066**	**1**	5	**1**	5	7	55	10	36	51	81	5	2	2	**1**
**BC067**	81	688	16	55	9	17	**1**	10	49	47	3	**1**	43	20
**SA068**	**1**	**1**	**1**	**1**	15	356	6	3	52	234	40	5	4	2
**SA070**	**1**	3	**1**	3	40	347	3	8	30	31	28	5	6	**1**
**SA072**	**1**	6	**1**	5	30	477	4	20	168	185	10	**1**	5	2
**SA076**	**1**	3	**1**	3	24	514	7	19	42	118	5	**1**	4	14

[£]Two first columns are for the vSAG 37-F6, which is the most recruiter virus in 13 of the 24 viromes and in the global marine virome. *Viromes used are abbreviated as: Pacific Ocean (POV), Chile-Peru oceanic region (CP), South Atlantic (SS), Red Sea (RS), Mediterranean Sea (MS), Northwest Arabian Sea upwelling (NAS), Indian Monsoon gyre province (IM), Eastern Africa Coastal Province (EA), Benguela Current (BC), and Sargassos Sea (SS), and the Blanes Bay Microbial Observatory Virome (BBMO) which was constructed in this study. [±]Viral genomic dataset used were: 40 marine surface vSAGs (this study), SAGs: 20 viral genomes from uncultured single bacterial cells; Isolates: 180 reference marine virus isolates (Supplementary Table 9), Fosmids: 1148 viral fosmids; TOV: 5466 viral contigs from the *Tara* expedition; and GOV: 3594 sequences from the cosmopolitan viral clusters previously described (VCs 2,3,5,6,8 and 9)[11].

Supplementary Table 6. Comparison by BLASTn of genome of vSAG 37-F6 with the previously described viral cluster 8 (VC_8; in this study VC_2)[11*]

Name	Bit-Score	Pairwise Identity	E Value	Hit end	Hit start	Query end	Query start
unknown_gi_486908286 (SAG AAA164-I21)	1353.81	70	0	612	3610	13588	10602
unknown_gi_486908286 (SAG AAA164-I21)	1142.82	76	0	8279	9887	5065	3480
Flavobacteriia_gi_487372893 (SAG AAA160-P02)	1092.32	72	0	32034	29947	12683	10605
GOV_bin_5468_contig-100_39	966.089	71	0	4638	2613	12620	10604
GOV_bin_2346_contig-100_4	933.628	71	0	1790	3825	12620	10603
GOV_bin_2164_contig-100_0	904.774	70	0	4841	6998	12975	10824
Flavobacteriia_gi_487372893 (SAG AAA160-P02)	839.853	72	0	25190	23594	5070	3479
GOV_bin_4626_contig-100_1	791.162	72	0	7594	6024	5082	3517
GOV_bin_2346_contig-100_4	751.488	71	0	8610	10209	5078	3483

*Only top ten best hits are shown

Supplementary Table 7. Comparison of metaproteomic data from the Oregon coast bacterioplankton[13] to our surface vSAG.

Peptide name[α]	Amino acid sequence	vSAG[β]
8431	YTVYKNPYMTENVILMGYK	37-F16
6640	TAMEGDFDTGNVR	37-F6
6420	SQLVKELEPGLNALFGLEYK	37-F6
5051	MIIPSELQFTAER	37-F6
4982	MFNRAPLTTAMEGDFDTGNVR	37-F6
1627	ELEPGLNALFGLEYK	37-F6
6422	SQLVKELEPGLNALFGLEYKR	37-F6
2780	QLVKELEPGLNALFGLEYK	37-F6
6706	TETYRDPDSFADIVR	37-H5 contig 2
7662	VLLCDEFATPAVSK	37-I21
4739	LSGEIGQVFGSR	37-I21
4493	LISQSYLGNETEEDAIMPILPLIR	37-I21
4022	KLISQSYLGNETEEDAIMPILPLIR	37-I21
3356	IGFTDLIDGATSK	37-I21
2454	GIENAILAGDDADGVYGTSGAAFEGLLHLAR	37-I21
1336	DIENELVLAPLFR	37-I21
5495	NLDKQGAIEENMLFLSR	37-J6 contig 1
5295	MVGAEMPMTSDQVIWSEQNR	37-J6 contig 1
4530	LLDEQNIPEEGR	37-K7 contig 3
4739	LSGEIGQVFGSR	37-M19
6387	SPIKTSMEGDFDTGNVR	41-A4 contig 1
5608	NQLVKELEPGLNALFGLEY	41-A4 contig 1
2780	QLVKELEPGLNALFGLEY	41-A4 contig 1
1627	ELEPGLNALFGLEY	41-A4 contig 1
912	QLVKELEPGLNALFGLEY	41-A4 contig 1
3786	ITGFADMIQLTHLK	41-D7 contig 1
2932	GVIVPAGTSTVYDQQLGK	41-D7 contig 1
6666	TASGISMLMSAANGSIR	41-H16
8431	YTVYKNPYMTENVILMGYK	41-H4 contig 2

[β]tBLASTx comparison was done and only those peptides matching 100% identity and coverage were considered
[α]Peptide name nomenclature was as in the original article[13]. A total of 7151 distinct peptide sequences were obtained in that study.

Supplementary Table 8. Primers of vSAG 37-F6.

Primer pair	Name	Sequence	Minimum	Maximum	Length	Direction	Expected size
1	37F6_78 F	ACGGGTCCAACTGAACATCC	78	97	20	forward	639
1	37F6_716 R	TAGCAGAGGATGGGTCAGCT	697	716	20	reverse	
2	37F6_697 F	AGCTGACCCATCCTCTGCTA	697	716	20	forward	1062
2	37F6_1,758 R	TGTGGTTTCGGGTGATGGAG	1,739	1,758	20	reverse	
3	37F6_697 F	AGCTGACCCATCCTCTGCTA	697	716	20	forward	1166
3	37F6_1,862 R	TGGTAATGCAGGCGTCCTTT	1,843	1,862	20	reverse	
4	37F6_4,647 F	GCATCCTCTGATCCTGCTCC	4,647	4,666	20	forward	788
4	37F6_5,434 R	AGAACACAGGCTGAACCGAG	5,415	5,434	20	reverse	
5	37F6_6,849 F	TCCGACTGTATCACTCGGGT	6,849	6,868	20	forward	818
5	37F6_7,666 R	AGGTGGTGGACTGTGCAAAA	7,647	7,666	20	reverse	

Supplementary Table 9. Marine virus isolates used for fragment recruitment analyses. Genomes were obtained from Joint Genome Institute. All viruses labelled as marine origin were considered (as of date 21st, January, 2016).

Genome name IMG-JGI / Genbank ID	Number of genes	Sequence Length (bp)
Bacteriophage 11b: NC_006356	65	36012
Bacteriophage K139: NC_003313	44	33106
Bacteriophage S-PM2 virion: NC_006820	264	196280
Bacteriophage Syn9 virus: NC_008296	235	176847
Bacteriophage VfO3K6: NC_002362	10	8784
Bacteriophage VfO4K68: NC_002363	8	6891
Cellulophaga phage phi10:1 / NC_021802	108	53664
Cellulophaga phage phi12:1 / NC_021791	64	39148
Cellulophaga phage phi12:2 / NC_021797	13	6453
Cellulophaga phage phi12a:1 / NC_021805	13	6478
Cellulophaga phage phi13:2 / NC_021803	128	72369
Cellulophaga phage phi14:2 / NC_021806	133	100418
Cellulophaga phage phi17:1 / NC_021795	65	38776
Cellulophaga phage phi17:2 / NC_021798	221	145343
Cellulophaga phage phi18:1 / NC_021790	65	39189
Cellulophaga phage phi18:3 / NC_021794	123	71443
Cellulophaga phage phi19:1 / NC_021799	118	57447
Cellulophaga phage phi3:1 / Ga0039577_11	36	22893
Cellulophaga phage phi38:1 / NC_021796	117	72534
Cellulophaga phage phi39:1 / NC_021804	48	28760
Cellulophaga phage phi4:1 / NC_021788	221	145865
Cellulophaga phage phi46:1 / NC_021800	54	34844
Cellulophaga phage phi46:3 / NC_021792	121	72961
Cellulophaga phage phi47:1 / HQ670749	81	60552
Cellulophaga phage phi48:2 / NC_021793	29	11703
Cellulophaga phage phiSM / HQ317392	59	44557
Cellulophaga phage phiST / Ga0040773_11	109	79114
Cyanophage 9515-10a / Ga0034026_11	62	47055
Cyanophage KBS-P-1A / Ga0032521_11	63	45730
Cyanophage KBS-S-1A / Ga0032522_11	60	32402
Cyanophage KBS-S-2A / Ga0039582_11	62	40658
Cyanophage MED4-117 / Ga0039388_11	66	38834
Cyanophage NATL1A-7 / Ga0034027_gi310005689.1	74	47741
Cyanophage NATL2A-133 / Ga0034029_gi310005755.1	73	47536
Cyanophage P60: NC_003390	80	47872
Cyanophage PP / NC_022751	41	42480
Cyanophage P-RSM1 / HQ634175	215	177211
Cyanophage P-RSM3 / HQ634176	211	178750

Cyanophage P-RSM6 / Ga0040776_11	229	192497
Cyanophage P-SS1 / Ga0040801_11	223	178284
Cyanophage PSS2 / GU071090	122	105532
Cyanophage PSS2: NC_013021	131	107530
Cyanophage P-SSM2: NC_006883	330	252401
Cyanophage P-SSM4: NC_006884	198	178249
Cyanophage P-SSP2 / Ga0034028_gi310005818.1	59	45890
Cyanophage P-SSP7: NC_006882	53	44970
Cyanophage SS120-1 / HQ316584	53	46997
Cyanophage S-SSM2 / Ga0032571_11	209	179980
Cyanophage S-SSM6a / HQ317391	311	232883
Cyanophage S-SSM6b / HQ316603	221	182368
Cyanophage S-TIM5 / NC_019516	190	161440
Cyanophage Syn10 / Ga0040497_11	219	177103
Cyanophage Syn2 / Ga0032453_11	218	175596
Cyanophage Syn30 / Ga0032525_11	225	178807
Cyanophage Syn5: NC_009531	61	46214
Emiliania huxleyi virus 86	477	407339
Flavobacterium phage 6H / NC_021867	63	46978
Marine bacteriophage RNA virus SOG	3	4449
Marine birnavirus - AY-98 VP1 / AY123970.1	1	2778
Marine gokushovirus	6	4129
Marine RNA virus JP-A	2	9236
Marine RNA virus JP-B	2	8926
Marinomonas phage P12026	54	31766
Ostreococcus lucimarinus virus OlV1	255	194022
Ostreococcus lucimarinus virus OlV3	265	191242
Ostreococcus lucimarinus virus OlV5	265	186468
Ostreococcus tauri virus 1	232	191761
Ostreococcus tauri virus 2	237	184409
Ostreococcus virus OsV5	269	185373
Paracoccus phage vB_PmaS_IMEP1 / Ga0062596_vB_PmaS_IMEP1.1	55	42093
Pelagibacter phage HTVC008M / NC_020484	198	147284
Pelagibacter phage HTVC010P / NC_020481	64	34892
Pelagibacter phage HTVC011P / NC_020482	45	39921
Pelagibacter phage HTVC019P / NC_020483	59	42084
Prochlorococcus phage MED4-184 / Ga0032523_11	65	38327
Prochlorococcus phage MED4-213 / HQ634174	218	180977
Prochlorococcus phage P-GSP1 / HQ332140	53	44945
Prochlorococcus phage P-HM1: NC_015280	241	181044
Prochlorococcus phage P-HM2: NC_015284	242	183806
Prochlorococcus phage P-RSM4: NC_015283	242	176428

Prochlorococcus phage P-RSP2 / HQ332139	48	42257
Prochlorococcus phage P-SSM2 / GU071092	332	252407
Prochlorococcus phage P-SSM3 / Ga0032395_11	231	179063
Prochlorococcus phage P-SSM5 / HQ632825	331	252013
Prochlorococcus phage P-SSM7: NC_015290	241	182180
Prochlorococcus phage P-SSP10 / Ga0039583_11	61	47325
Prochlorococcus phage P-SSP3 / HQ332137	56	46198
Prochlorococcus phage P-SSP7 / GU071093	52	45135
Prochlorococcus phage Syn1: NC_015288	240	191195
Prochlorococcus phage Syn33: NC_015285	232	174285
Pseudoalteromonas phage PSA-HS4 (complete) / Ga0074570_11	68	38739
Puniceispirillum phage HMO-2011	43	52512
Roseobacter phage RDJL Phi 1: NC_015466	87	62668
Roseophage SIO1: NC_002519	34	39898
Synechococcus phage KBS-M-1A / Ga0039581_11	226	171744
Synechococcus phage metaG-MbCM1 / NC_019443	234	172879
Synechococcus phage S-CAM1 / HQ634177	241	198013
Synechococcus phage S-CAM8 / Ga0039739_11	277	222057
Synechococcus phage S-CAM8 / HQ634178	209	171407
Synechococcus phage S-CBM2 / HQ633061	212	180892
Synechococcus phage S-CBP2 / Ga0032396_11	137	92473
Synechococcus phage S-CBP3 / HQ633062	57	47375
Synechococcus phage S-CBP4 / Ga0039743_11	57	41824
Synechococcus phage S-CBS1 / Ga0035795_11	47	30332
Synechococcus phage S-CBS2: NC_015463	102	72332
Synechococcus phage S-CBS3: NC_015465	46	33004
Synechococcus phage S-CBS4 / Ga0035827_gi374531742.1	108	69420
Synechococcus phage S-CBS4 / HQ634148	167	105580
Synechococcus phage S-CRM01: NC_015569	330	178563
Synechococcus phage S-IOM18 / HQ317383	219	171797
Synechococcus phage S-MbCM6 / NC_019444	225	176043
Synechococcus phage S-RIM2 R1_1999 / HQ317292	216	175430
Synechococcus phage S-RIM2 R21_2007 / HQ317290	214	175430
Synechococcus phage S-RIM2 R9_2006 / HQ317291	217	175419
Synechococcus phage S-RIM8 A.HR1 / Ga0039740_gi375918176.1	225	171211
Synechococcus phage S-RIM8 A.HR3 / Ga0032513_gi375919032.1	225	171211
Synechococcus phage S-RIM8 A.HR5 / HQ317385	211	168327
Synechococcus phage S-RIP1 / HQ317388	61	44892
Synechococcus phage S-RIP2 / HQ317389	57	45728
Synechococcus phage S-RSM4: NC_013085	249	194454
Synechococcus phage S-ShM2: NC_015281	231	179563

Synechococcus phage S-SKS1 / HQ633071	302	208007
Synechococcus phage S-SM1: NC_015282	240	174079
Synechococcus phage S-SM2: NC_015279	278	190789
Synechococcus phage S-SSM4 / HQ316583	223	182801
Synechococcus phage S-SSM5: NC_015289	229	176184
Synechococcus phage S-SSM7: NC_015287	324	232878
Synechococcus phage Syn19: NC_015286	221	175230
Vibrio cholerae filamentous bacteriophage fs-2: NC_001956	9	8651
Vibrio cholerae O139 fs1 phage: NC_004306	15	6340
Vibrio cholerae phage KSF-1phi virus: NC_006294	12	7107
Vibrio cholerae phage VGJphi virion: NC_004736	13	7542
Vibrio harveyi bacteriophage VHML: NC_004456	57	43198
Vibrio phage 11895-B1 / Ga0040774_11	206	126434
Vibrio phage CP-T1 / NC_019457	70	44492
Vibrio phage CTX chromosome I: NC_015209	13	10638
Vibrio phage douglas 12A4 / HQ316580	75	57611
Vibrio phage eugene 12A10 / HQ634195	253	138234
Vibrio phage helene 12B3 / HQ316579	265	135982
Vibrio phage henriette 12B8 / HQ316582	156	107218
Vibrio phage ICP1: NC_015157	230	125956
Vibrio phage ICP2: NC_015158	72	49675
Vibrio phage ICP3: NC_015159	54	39162
Vibrio phage JA-1 / NC_021540	80	69278
Vibrio phage jenny 12G5 / HQ632860	75	40557
Vibrio phage kappa: NC_010275	45	33134
Vibrio phage KVP40: NC_005083	410	244834
Vibrio phage martha 12B12 / HQ316581	51	33277
Vibrio phage N4: NC_013651	47	38497
Vibrio phage nt-1 / HQ317393	405	247511
Vibrio phage pVp-1 / NC_019529	157	111506
Vibrio phage PWH3a-P1 / Ga0039735_11	216	129155
Vibrio phage pYD21-A / Ga0032403_11	75	46917
Vibrio phage pYD38-A / Ga0032404_11	76	47552
Vibrio phage pYD38-B / Ga0040529_11	60	37324
Vibrio phage SIO-2 / HQ316604	116	81184
Vibrio phage vB_VchM-138 / NC_019518	67	44485
Vibrio phage vB_VpaM_MAR / NC_019722	62	41351
Vibrio phage vB_VpaS_MAR10 / NC_019713	107	78751
Vibrio phage VBM1 / HQ317386	56	38374
Vibrio phage VBP32 / Ga0032561_11	117	76718
Vibrio phage VBP47 / Ga0040770_11	119	76705
Vibrio phage VBpm10 / Ga0039578_11	62	33314

Vibrio phage VCY-phi / Ga0036010_11	11	7103
Vibrio phage VD1 / Ga0032407_11	116	81013
Vibrio phage VEJphi: NC_012757	11	6842
Vibrio phage Vf12: NC_005949	7	7965
Vibrio phage Vf33: NC_005948	7	7965
Vibrio phage VFJ / NC_021562	12	8555
Vibrio phage VP882: NC_009016	71	38197
Vibrio phage VP93: NC_012662	44	43931
Vibrio phage VPMS1 / NC_021776	53	42313
Vibrio phage VPUSM 8 / NC_022747	43	34145
Vibrio phage VSK: NC_003327	14	6882
Vibriophage VP2: NC_005879	47	39853
Vibriophage VP4: NC_007149	31	39503
Vibriophage VP5: NC_005891	48	39786
Vibriophage VpV262: NC_003907	67	46012
Yellowtail ascites virus strain AY-98 segment A / AY283785	2	3092

Supplementary Notes

Supplementary Note 1: Fluorescence activated virus sorting (FAVS) and whole genome amplification (WGA): some technical considerations

Viruses are sorted at random, which means that the more abundant a virus is in the sample the higher is the probability to be sorted and deposited in a 384-well plate, and thus, is directly proportional to its abundance. Assuming that the treatment to break capsids is effective to most naturally co-occurring viruses, in theory, with a low sequencing effort, SVGs guarantees the recovering of genetic information of prevalent viral components. Furthermore, as sequencing costs has dropped dramatically in the last five years along with new inexpensive multiplexed libraries strategies[17] and the fact that the sequencing coverage for a virus is significantly less than for a single-cell, genome recovery of low abundant viruses by increasing the number of positive vSAGs selected for sequencing should be feasible.

Supplementary Note 2: Evaluation of free DNA content in microdroplets from seawater

Initially, the interference of free DNA present in seawater that could be co-sorted along with single-viruses and amplified during WGA was assessed (see methods), but data indicated that its potential contribution was negligent (Supplementary Fig. 4d-e) since only two wells from a 384-well plate yielded positive amplification.

Supplementary Note 3: Gene-content based network analysis of marine vSAGs

Of the 61 marine vSAG sequences, 57 were retained in the network and 4 (17-C23-contig2, 17-F19-contig3, 37-K7-contig1, 37-L15-contig3) were excluded, due to few significant similarities to other sequences in the dataset. In cases where a vSAG consisted of several sequences (e.g. 17-F19, 37-K7, 41-H4), vSAG fragments were mostly associated within the same viral clusters (VCs) in GOV[11], expect in some cases where small contigs were obtained along with the large genome fragment, such as the vSAG 17-F19-contig1 (15,706 bp) and contig2 (2,525 bp) that were related to members of VC13, whereas contig3 (2,236 bp) was not found within that network. In cases where disagreements exist, it is highly likely that each sequence fragment carries a different set of gene sequences less related to genes on its sister fragment than to genes present on sequences in separate VCs. The 57 sequences were related to a total of 31 VCs. The VCs ranged in size from 2 (VC_733) to 1090 (VC_0), with most vSAGs associated with large (>100 sequence) VCs. The 19 vSAGs identified through comparison using BLASTn (Supplementary Table 4) covered 14 of the GOV-associated VCs. Disagreements between the BLASTn and network analysis could arise from the differences in approaches, where BLASTn tends to reveal highly related sequences though pairwise relationships whereas the gene-based method allows for sequences to associate with multiple others, with sequences sharing the greatest proportion of genes being placed within the same cluster. In general, the larger the VC the more likely it contained a GOV-associated VC and agreed with BLAST. Due to the inclusion of archaeal and bacterial viruses from NCBI RefSeq, preliminary taxonomic predictions could be made in the context of reference sequences within each VC. Tentative affiliations could only be made for 24 of the 57 sequences (21 vSAGs) due to the lack of any reference sequence within

the VCs (Supplementary Table 3). All taxonomic predictions were of the *Caudovirales*, with 18 sequences (15 vSAGs) classified in the *Podoviridae* family, 3 sequences (3 vSAGs) as *Myoviridae* and 3 sequences (3 vSAGs) as *Siphoviridae*. The overall prediction quality of all but 2 sequences (17-C23-contig1, 30-E13) were low, as most of the VCs containing reference sequences were supported by 1-2 references within VCs containing 130 to over 200 sequences. The strongest support was for vSAG 17-C23-contig1 and 30-E13, both members of VC_78 and likely T5-like viruses.

Supplementary Note 4: Virome recruitment of marine single amplified viral genomes (vSAGs)

In this study, with 44 surface vSAGs that added up to ≈1 Mb of genomic assembled dataset (<5 million raw reads), we have unveiled the genome of superabundant uncultured viruses with very high virome recruitment frequencies. In the *Tara* virome survey[7], with 5,476 viral contigs (109 Mb of assembled genome data and 2,16 billion raw reads) recruited up to 9.97%[7]. However, after normalization of recruitment rate according to total assembled genomic data, 1 Mb of single-virus genomic data would recruit ≈3.5-fold more than data obtained by viromics (Supplementary Fig. 13). Finally, the overall sequencing effort carried out here to deliver 44 reference genomes compared to previous viromic surveys[7] was significantly less, at least a 3-fold decrease.

Supplementary Note 5: Structure of marine viral populations. Microdiversity matters for metagenomic assembly: the diversity curves

The diversity curves that represent the relative distribution of recruited reads at different nucleotide identities for a given viral reference genome in a virome informs about the structure and (micro)-diversity of a particular viral population at the species and genus level. In general, for most vSAGs and reference virus isolates showed a unimodal pattern in the diversity curve with a recruitment peak of recruited read frequency near 90% of identity and no recruitment was observed below 75% of identity. To summarize, we propose a model based on our obtained diversity curves that is depicted in Supplementary Fig 10c:

1) In general, the more (micro-) diverse is a viral population, the lower is the height of the curve (value H), and the higher is the width of the curve (value W) (Supplementary Fig 10c). In contrast, in a scenario where an abundant virus has no viral relatives co-existing in the same population (no microdiversity), the pattern of its viral population structure would be a narrow sharp curve, such as the metagenomic contigs depicted in Fig. 6b.

2) Recruited reads with identity values around 95% or higher were likely from our reference vSAG and/or close viral relatives belonging mostly to the same population at species-level.

3) Recruited reads with identity values under the observed empirical peak around 90% are from viral relatives belonging mostly to the same population at the genus or sub-family levels.

As shown in Fig. 6a, single-virus genomic approach can uncover the reference genome of uncultured viral populations regardless of the accumulated microdiversity since the complexity in terms of genome reconstruction is simplified. For viromics, in

general (Fig. 6a), we have observed that the species-specific recruitment patterns for many of the most abundant assembled genomes (viral contigs) in their own *Tara* viromes lacked of microdiversity. We analyzed over 50 abundant viral species (Fig. 6a; for convenience only 12 are shown in that panel) obtained from *Tara dataset* in different oceanic regions, and overall, the obtained pattern suggested a lack of microdiversity in these viral species populations at the sampling site where they were generated. This likely means, as we demonstrated in our simulated viromes (Fig. 6c, Supplementary Fig 20), that the assembler resolved successfully the genome reconstruction only for those populations mostly when the microdiversity scenario was low and there was sufficient sequencing coverage to be assembled; in other words, overall fairly abundant in the viral community and very dominant within its population. In turn, for those highly microdiverse and diverse populations, despite they are abundant, the assembler yielded small genome fragments and a very partial reconstruction. In the case of the *Tara* expedition[32], where MOCAT assembler was used, all obtained diversity curves for assembled viral contigs that were abundant in the corresponding viral assemblages lacked of microdiversity, except in two viral contigs (22SUR_22922 and 64SUR_1238) where the observed species-specific recruitment pattern indicated low microdiversity. In our study, with our virome from Blanes, we have observed that with IDBA_UD and SPAdes assemblers, in some cases, they delivered viral contigs representing viral populations with moderate microdiversity. Thus, the selection of the metagenomic assembler could have a negative impact on the genomic reconstruction, biasing thus the biological conclusions. We suggest from our analyses, that SPAdes could outperform other programs in terms of resolving the genome reconstruction from microdiverse viral populations.

Finally, it is important to remark that nearly all diversity curves obtained for the tested reference viral isolates, fosmids, vSAGs and viruses found in single-cells for all studied viromes (Supplementary Fig 10) showed that viral populations in general tend to be structured accumulating diversity and microdiversity. Therefore, the fact of finding diversity curves lacking of microdiversity when a viral contig “X” is compared against its own virome “X”, shows:

1) that virus “X” clearly bloomed in that specific virome “X” dominating its population over other viral relatives belonging to same population (e.g. kill the winner scenario)

2) the inability of the assembler in general to resolve the assembly from highly microdiverse and diverse viral populations regardless the abundance. In fact, for many cases where a particular viral contig in its own virome showed a diversity curve lacking of microdiversity (e.g. above case of virus “X”), when it was computed for other viromes (Y, Z, etc…), the curve revealed the existing microdiversity of that population, indicating likely that in these other virome samples, that particular virus “X” was not dominating the population. However, we hypothesize that from the later virome sample (virome Y or Z), where dominance of the virus X was not observed; likely the genome of virus X would not be reconstructed by assemblers such as MOCAT.

Supplementary Methods

Simulation of natural viromes with different degrees of microdiversity

Firstly, we selected the *Tara* virome MS022[7] from the Mediterranean Sea for our simulation as a model since we previously demonstrated by fragment recruitment and diversity curves the presence of the highly microdiverse population of vSAG 37-F6. It is worth noting that in a previous study[7], from this natural virome dataset, MOCAT assembler was unable to reconstruct the genome of virus vSAG 37-F6 despite its abundance. Later, with the same dataset, by using IDBA_UD, which in principle outperforms MOCAT assembler, combined with genome binning[11] failed on the genome reconstruction of virus 37-F6. From that *Tara* virome MS022 dataset, we subtracted the raw reads corresponding with 37-F6 virus population. For that, we mapped the whole *Tara* virome MS022 against reference virus vSAG 37-F6 and a total of 74,278 reads were removed from the dataset. Geneious bioinformatic program[18] was used to map and subtract the reads with the parameters previously used for fragment recruitment (identity >70% and mean coverage >90%). Supplementary Fig. 20d shows that no reads belonging to 37-F6 population remained in the dataset. The trimming tool Trimmomatic version 0.36 was used to ensure that all remained reads in the virome were in the paired-end format for the metagenomic assembly after removing reads corresponding to vSAG 37-F6 population. Then, taking the reference genome vSAG 37-F6, we simulated three scenarios with different populations, A, B and C with different degrees of microdiversity and diversity (Supplementary Fig. 20b). Population A has no microdiversity with two simulated genomes (genome of vSAG 37-F6 and a simulated genome 1 with >99.9% nucleotide identity) and only 20 SNPs of difference. Population B is a low microdiverse population with 5 simulated genomes with approximately ≥95% nucleotide genome identity along all genome including in the hypervariable genome island (Fig. 4 and Supplementary Fig. 14). This is likely a simplistic scenario since in many cases even close viral relatives have a large variability in the hypervariable genomic island[19]. Population C is a medium-high microdiverse population with 10 simulated genomes. Eight of which had approximately ≥90% nucleotide genome identity along all genome, except in the genomic island, where higher genetic variability was introduced among the simulated genomes with <50% nucleotide identity in that region. The global nucleotide identity value of 90% was taken from the empirical peak observed in the resulting diversity curves for the natural population of vSAG 37-F6 in *Tara* MS022 virome (Supplementary Fig. 10). The value of 50% of identity for the genomic island has been taken according to the recruitment plot obtained for vSAG 37-F6 in different viromes where very high variability was observed. In addition, existing data on the co-existence of several virus isolate strains with high global genome identity but high variability in the genomic islands are described[19]. The remaining two simulated genomes (no. 7 and 9) were genetically more distant with the rest of genomes, approximately 80% identity value. The genomes were simulated with the publicly available bioinformatic tool at the following link: http://www.bioinformatics.org/sms2/mutate_dna.html. Then, with these simulated genomes for each population and assuming equal abundance of each genome within the population, we generated approximately a total of 74,278 Illumina reads for each population by using the program Art[20] that can simulate the same Illumina error rate

for the HiSeq 2000 platform previously used to sequence the *Tara* virome dataset. The parameters used were art_illumina -ss HS20 -sam -p -l 100 -s 10 -o paired_dat. (Supplementary Fig. 20c). Those simulated reads from each one of the populations were merged with the *Tara* MS022 virome where reads of 37-F6 were removed (Supplementary Fig. 20d). So, three different *Tara* MS022 viromes were finally constructed with different and controlled degrees of microdiversity, (Supplementary Fig. 20e) in which the reference genomes forming that population were known. Finally, these three simulated natural viromes were assembled by IDBA_UD with the same parameters previously used (--mink 20 –maxk 100 –step 20 –min_contig 1000) and described for that virome reconstruction[11]. In addition, SPAdes[21] version 3.9 was used with the following parameters for metagenomic assembly: "metaspades.py -k 33,55,77,99". Obtained contigs were mapped against the simulated reference genomes for each one of the population with the following cut-off parameters: ≥95% of identity value and ≥80% of contig coverage.

Supplementary References

1. Brussaard, C. P. D. Optimization of Procedures for Counting Viruses by Flow Cytometry. *Appl. Environ. Microbiol.* **70,** 1506–1513 (2004).

2. Tennessen, K. *et al.* ProDeGe: a computational protocol for fully automated decontamination of genomes. *ISME J.* **10,** 269–272 (2015).

3. Mills, R., Rozanov, M., Lomsadze, A., Tatusova, T. & Borodovsky, M. Improving gene annotation of complete viral genomes. *Nucleic Acids Res.* **31,** 7041–7055 (2003).

4. Besemer, J. & Borodovsky, M. GeneMark: Web software for gene finding in prokaryotes, eukaryotes and viruses. *Nucleic Acids Res.* **33,** 451–454 (2005).

5. Mizuno, C. M., Ghai, R., Saghaï, A., López-García, P. & Rodriguez-Valera, F. Genomes of abundant and widespread viruses from the deep ocean. *MBio* **7,** e00805-16 (2016).

6. Marchler-Bauer, A. *et al.* CDD: a Conserved Domain Database for the functional annotation of proteins. *Nucleic Acids Res.* **39,** D225-9 (2011).

7. Brum, J. R. *et al.* Ocean plankton. Patterns and ecological drivers of ocean viral communities. *Science* **348,** 1261498 (2015).

8. Caro-quintero, A. & Konstantinidis, K. T. Bacterial species may exist, metagenomics reveal. *Environ. Microbiol.* **14,** 347–55 (2012).

9. Labonté, J. M. *et al.* Single-cell genomics-based analysis of virus-host interactions in marine surface bacterioplankton. *ISME J.* **9,** 2386–2399 (2015).

10. Mizuno, C. M., Rodriguez-Valera, F., Kimes, N. E. & Ghai, R. Expanding the marine virosphere using metagenomics. *PLoS Genet.* **9,** e1003987 (2013).

11. Roux, S. *et al.* Ecogenomics and potential biogeochemical impacts of globally abundant ocean viruses. *Nature* **537,** 689–693 (2016).

12. Brum, J. R. *et al.* Illuminating structural proteins in viral 'dark matter' with metaproteomics. *Proc. Natl. Acad. Sci. U. S. A.* **113,** 2436–2441 (2016).

13. Sowell, S. M. *et al.* Environmental proteomics of microbial plankton in a highly productive coastal upwelling system. *ISME J.* **5,** 856–65 (2011).

14. Zhao, Y. *et al.* Abundant SAR11 viruses in the ocean. *Nature* **494,** 357–360 (2013).

15. Hurwitz, B. L. & Sullivan, M. B. The Pacific Ocean Virome (POV): a marine viral metagenomic dataset and associated protein clusters for quantitative viral ecology.

PLoS One **8,** (2013).

16. Angly, F. E. *et al.* The marine viromes of four oceanic regions. *PLoS Biol.* **4,** e368 (2006).

17. Baym, M. *et al.* Inexpensive multiplexed library preparation for megabase-sized genomes. *PLoS One* **10,** 1–15 (2015).

18. Kearse, M. *et al.* Geneious Basic: an integrated and extendable desktop software platform for the organization and analysis of sequence data. *Bioinformatics* **28,** 1647–9 (2012).

19. Mizuno, C. M., Ghai, R. & Rodriguez-Valera, F. Evidence for metaviromic islands in marine phages. *Front. Microbiol.* **5,** (2014).

20. Huang, W., Li, L., Myers, J. R. & Marth, G. T. ART: a next-generation sequencing read simulator. *Bioinformatics* **28,** 593–4 (2012).

21. Bankevich, A. *et al.* SPAdes: a new genome assembly algorithm and its applications to single-cell sequencing. *J. Comput. Biol.* **19,** 455–77 (2012).
